# Effect of Enhanced Medical Rehabilitation on Functional Recovery in Older Adults Receiving Skilled Nursing Care After Acute Rehabilitation: A Randomized Clinical Trial

**DOI:** 10.1001/jamanetworkopen.2019.8199

**Published:** 2019-07-31

**Authors:** Eric J. Lenze, Emily Lenard, Marghuretta Bland, Peggy Barco, J. Philip Miller, Michael Yingling, Catherine E. Lang, Nancy Morrow-Howell, Carolyn M. Baum, Ellen F. Binder, Thomas L. Rodebaugh

**Affiliations:** 1 Healthy Mind Lab, Department of Psychiatry, Washington University School of Medicine in St Louis, St Louis, Missouri; 2 Program in Physical Therapy, Washington University School of Medicine in St Louis, St Louis, Missouri; 3 Program in Occupational Therapy, Washington University School of Medicine in St Louis, St Louis, Missouri; 4 Division of Biostatistics, Washington University School of Medicine in St Louis, St Louis, Missouri; 5 Brown School of Social Work, Washington University in St Louis, St Louis, Missouri; 6 Division of Geriatrics and Nutritional Science, Washington University School of Medicine in St Louis, St Louis, Missouri; 7 Department of Psychological and Brain Sciences, Washington University in St Louis, St Louis, Missouri

## Abstract

**Importance:**

Enhanced medical rehabilitation (EMR) is a systematic and standardized approach for physical and occupational therapists to engage patients. Higher patient engagement in therapy might lead to improved functional recovery in rehabilitation settings, such as skilled nursing facilities (SNFs).

**Objective:**

To determine whether EMR improves older adults’ functional recovery.

**Design, Setting, and Participants:**

A double-blind, parallel-group, randomized clinical trial was conducted from July 29, 2014, to July 13, 2018, in 229 adults aged 65 years or older admitted to 2 US SNFs. Participants were randomized to receive EMR (n = 114) vs standard-of-care rehabilitation (n = 115). Intention-to-treat analysis was used.

**Interventions:**

The intervention group received their physical and occupational therapy from therapists trained in EMR. Based on models of motivation and behavior change, EMR is a toolkit of techniques to increase patient engagement and therapy intensity. The control group received standard-of-care rehabilitation from physical and occupational therapists not trained in EMR.

**Main Outcomes and Measures:**

The primary outcome was change in function in activities of daily living and mobility, as assessed with the Barthel Index, which measures 10 basic activities of daily living or mobility items (scale range, 0-100), from SNF admission to discharge; secondary outcomes were gait speed for 10 m, 6-minute walk test, discharge disposition, rehospitalizations, and self-reported functional status at days 30, 60, and 90. To examine the rehabilitation process, therapists’ engagement with patients and patient active time during therapy were measured for a sample of the sessions.

**Results:**

Of the 229 participants, 149 (65.1%) were women; 177 (77.3%) were white, and 51 (22.3%) were black; mean (SD) age was 79.3 (8.0) years. Participants assigned to EMR showed greater recovery of function than those assigned to standard of care (mean increase in Barthel Index score, 35 points; 95% CI, 31.6-38.3 vs 28 points; 95% CI, 25.2-31.7 points; *P* = .007). There was no evidence of a difference in the length of stay (mean [SD], 23.5 [13.1] days). However, there were no group by time differences in secondary outcome measures, including self-reported function after SNF discharge out to 90 days as measured on the Barthel Index (mean [SE] score: EMR, 83.65 [2.20]; standard of care, 84.67 [2.16]; *P* = .96). The EMR therapists used a median (interquartile range) of 24.4 (21.0-37.3) motivational messages per therapy session vs 2.3 (1.1-2.9) for nontrained therapists (*P* < .001), and EMR patients were active during a mean (SD) of 52.5 (6.6%) of the therapy session time vs 41.2 (6.8%) for nontrained therapists (*P* = .001).

**Conclusions and Relevance:**

Enhanced medical rehabilitation modestly improved short-term functional recovery for selected older adults rehabilitating in SNFs. However, there was no evidence that the benefits persisted over the longer term. This study demonstrates the value of engaging and motivating older adults in rehabilitation therapy, but more work is needed to extend these benefits to longer-term outcomes after discharge home.

**Trial Registration:**

ClinicalTrials.gov identifier: NCT02114879

## Key Points

**Question:**

Does enhanced medical rehabilitation, a technique for systematically engaging and motivating patients in their physical and occupational therapy, result in better functional outcomes?

**Findings:**

In this randomized clinical trial of 229 older adults receiving physical and occupational therapy in skilled nursing facilities, those receiving enhanced medical rehabilitation showed a higher percentage of active time during therapy sessions and a 25% greater functional recovery compared with those receiving standard-of-care therapy.

**Meaning:**

Systematically engaging older adults in their therapy after acute rehabilitation may result in a better functional outcome.

## Teaser

This randomized clinical trial examines whether an enhanced medical rehabilitation intervention, a systematic approach for physical and occupational therapy to engage patients, improves functional recovery in older patients receiving skilled nursing care after acute rehabilitation.

## Introduction

Older adults who experience a disabling medical event, such as hip fracture, require physical and occupational therapy (PT/OT) in postacute care settings, such as skilled nursing facilities (SNFs). The use of postacute care has grown as frail and medically complex older adults survive medical events but with such functional incapacity that they are unable to return home and function independently.^1,2^ In 2016, Medicare paid approximately $60 billion for postacute care, including 2.4 million SNF stays.^3^ For such patients, postacute rehabilitation is a window of opportunity to regain functional ability. The alternative is persistent disability, which comes with considerable human costs, as well as high health care costs,^4^ much of it resulting from rehospitalizations.^5,6,7,8^

To date, efforts to improve rehabilitation outcomes of older adults have met with limited success in randomized clinical trials.^9,10^ Increasing intensity by providing more PT/OT time in SNF therapy has modestly improved functional gains^11,12,13,14^ and increased the rate of discharge to community settings.^15^ Yet, a further increase in the use of therapy is likely not an option owing to cost-containment considerations.^16,17^ Instead, postacute rehabilitation could be optimized by improving therapists’ engagement with patients and the intensity of the therapy sessions, resulting in greater patient active time or patient activity per minute of PT/OT.^4,18,19,20,21,22,23,24^ Such engagement efforts must account for patient factors, such as depression, cognitive impairment, and multiple medical comorbidities, that can undermine motivation.^25,26,27,28^

Therefore, enhanced medical rehabilitation (EMR), a set of techniques and tools for therapists to engage patients in therapy, was developed.^29,30^ Enhanced medical rehabilitation is a systematic and standardized approach based on behavior change principles^31,32,33,34^ to enhance patient engagement and intensity to promote optimal functional outcomes. Enhanced medical rehabilitation was designed for real-world therapists and uses short, intuitive motivational messages and simple tools to help the therapist link PT/OT activities, effort, and progress to attainment of the patient’s selected and personally meaningful goals (Table 1).^35^

**Table 1.** Enhanced Medical Rehabilitation Tools^a^

Tool	Description	Guidelines for Use	Objective
**Patient Engagement Tools**			
Personal goals interview	Card sort of pictures of common activities older adults enjoy	Therapist instructs patient to sort cards into activities that are most important to them (vs less important)	Determine individualized goals to personalize therapy and increase patient’s motivation
Therapy tracker	Individualized patient brochure of goals, activities, and progress	Therapist records patient priority goals and activities needed to reach those goals with each patient; patient-friendly progress charts are developed by the therapist on the therapy tracker; the therapy tracker is shown to patient before and after therapy activities	Visually depict how each activity performed in a therapy session relates to a patient’s goal
Effort card	A card with a rating and description of effort levels from 1 (easy…I could be working much harder) to 5 (too hard… this is too hard for me)	Throughout treatment sessions, therapist asks patient to assess their effort; therapist provides positive reinforcement for achieving high effort and connects effort to achievement of personal goals	Visually demonstrate to the patient how much effort they are using and when they need to work harder, guiding the therapist in providing feedback to the patient and linking the patient’s effort toward reaching their personal goals
Home photograph guide	A small brochure that becomes individualized to each patient’s potential discharge environment	Significant others or family members compile and attach key photographs of the patient’s home (eg, number and depth of stairs, bed height, bathroom setup)	Ensure that the activities worked on in therapy directly transfer to the patient’s home or discharge environment
**Therapist Adherence Tools**			
Training	There are 5 formal training sessions with didactic and interactive methods. Training materials include a training manual and slide set.	The training summarizes all procedures and gives examples through interactive cases	Train and ensure high therapist adherence to EMR protocol and techniques
Coaching feedback form	A standardized checklist for the expert EMR coach is used to assess EMR techniques during an observed session and provide timely feedback to the therapist	Expert EMR therapist coach shadows with therapists in training and provides direct and timely feedback	Maintain high adherence to EMR techniques
Before, during, and after checklist	A standardized checklist is devoted to prompting the therapist to carry out the EMR steps and build self-awareness of the use of the EMR techniques	While the therapist is learning EMR, the checklist can be self-administered to help facilitate learning and follow through of the techniques (eg, how to respond to patient distress with empathy)	Help therapists attain and maintain high adherence to EMR techniques

Abbreviation: EMR, enhanced medical rehabilitation.

^a^ Tools and training are available at https://healthymind.wustl.edu/items/enhanced-medical-rehabilitation/.^35^

Preliminary research showed that PT and OT therapists could be trained to use EMR, resulting in higher patient active time and better functional outcomes.^30^ The purpose of the present study was to compare EMR with standard-of-care therapy for older adults receiving postacute rehabilitation in 2 area SNFs. We hypothesized that patients randomized to receive PT/OT from EMR-trained therapists would have better functional outcomes than those receiving standard-of-care therapy. We also examined whether patient characteristics—depression, cognitive impairment, and medical burden—influenced therapy outcome.

## Methods

### Participants

From July 29, 2014, to March 22, 2018, patients were recruited on admission to 2 SNFs in the St Louis, Missouri, metropolitan area. These facilities were selected based on their willingness to participate in the study and the number and diversity of patients admitted who were receiving postacute care. Participant inclusion criteria were age 65 years or older, admitted from an acute care hospital (Table 2), and requiring 2 or more weeks of rehabilitation with the potential to return to the community; individuals already residing in long-term care facilities before hospitalization were excluded. Other exclusion criteria were language, visual, or hearing barriers to participation; medical illness preventing study participation (including metastatic cancer, ongoing cancer treatment, hemodialysis, hospice care, or highly unstable cardiac illnesses with anticipated rehospitalization); moderate to severe cognitive impairment (demonstrated by medical record diagnosis of dementia and/or Short Blessed Test^36^ score >13); or psychotic disorder. The study was completed July 13, 2018.

**Table 2.** Baseline Characteristics of Participants

Characteristic	Group, No. (%)		
	Total (N = 229)	EMR (n = 114)	Standard of Care (n = 115)
Age, mean (SD), y	79.3 (8.0)	79.5 (8.2)	79.2 (7.7)
Sex			
Male	80 (34.9)	40 (35.1)	40 (34.8)
Female	149 (65.1)	74 (64.9)	75 (65.2)
Race/ethnicity			
White	177 (77.3)	88 (77.2)	89 (77.4)
Black	51 (22.3)	25 (21.9)	26 (22.6)
>1 Race	1 (0.4)	1 (0.9)	0 (0.0)
Hispanic or Latino	1 (0.4)	0	1 (0.9)
Not Hispanic or Latino	228 (99.6)	114 (100)	114 (99.1)
Primary impairment type			
Musculoskeletal/integument	80 (34.9)	36 (31.6)	44 (38.3)
Heart	60 (26.2)	31 (27.2)	29 (25.2)
Respiratory	43 (18.8)	25 (21.9)	18 (15.7)
Renal	14 (6.1)	7 (6.1)	7 (6.1)
Neurologic	10 (4.3)	5 (4.4)	5 (4.3)
Other/unknown	41 (17.9)	19 (17.4)	22 (19.8)
Depressive symptom severity: Montgomery-Äsberg Depression Rating Scale score, mean (SD)^a^	8.6 (7.8)	8.8 (7.6)	8.4 (8.0)
Cognitive impairment: Short Blessed Test score, mean (SD)^b^	4.1 (3.4)	4.4 (3.6)	3.8 (3.2)
Barthel Index total score, mean (SD)^c^			
Premorbid^d^	95.6 (8.1)	96.1 (6.9)	95.1 (9.0)
Admission^e^	33.5 (13.0)	32.3 (13.1)	34.7 (12.8)
Medical complexity: Cumulative Illness Rating Scale for Geriatrics score, mean (SD)^f^^,^^g^	16.9 (5.2)	16.8 (5.0)	17.1 (5.4)
Length of stay, mean (SD), d^h^	23.5 (13.1)	23.5 (14.4)	23.4 (11.7)

Abbreviation: EMR, enhanced medical rehabilitation.

^a^ Scores of 15 or higher indicate major depression.

^b^ Scores of 5 to 9 consistent with mild cognitive impairment and 10 or higher consistent with dementia.

^c^ Scale range, 0 to 100, with higher scores indicating greater levels of function.

^d^ The premorbid sample size was 227 (EMR, 113; standard of care, 114).

^e^ The sample size on admission was 228 (EMR, 114; standard of care, 114).

^f^ The sample size was 220 (EMR, 109; standard of care, 111).

^g^ Higher scores indicate greater comorbid burden.

^h^ The sample size was 221 (EMR, 111; standard of care, 110).

This study followed the Consolidated Standards of Reporting Trials (CONSORT) reporting guideline for randomized clinical trials. This study was a randomized clinical trial with 2 parallel groups (EMR vs standard of care) and blinded outcome assessments. The study was approved by the Washington University Institutional Review Board. Potential participants were enrolled after providing university-approved written informed consent. The trial protocol is available in Supplement 1.

### EMR Therapist Selection

Therapists were selected for EMR by measuring their pretraining patient active time and number of motivational messages and creating EMR and standard-of-care therapist groups who were equated in these variables (Table 3) as well as years and level of experience. Eleven therapists (occupational therapists, physical therapists, and certified therapy assistants) were trained in EMR, including 7 at the larger facility and 4 at the smaller one. Eighteen therapists were not trained and performed standard-of-care therapy. Therapist selection was conducted in collaboration with therapy managers to ensure adequate staffing coverage in each group (EMR and standard of care). Therapists had the opportunity to decline participation in the training and thus be in the standard-of-care group. To prevent bias or contamination between groups, the research team did not provide the standard-of-care therapists with information about EMR and discouraged the EMR-trained therapists from sharing it with them.

**Table 3.** Therapists’ Techniques Before and After Training in Enhanced Medical Rehabilitation

Therapist Technique or Process Evaluated	Pretraining				Posttraining (During Randomized Trial)^a^			
	EMR Therapists	Standard-of-Care Therapists	Analysis	*P* Value	EMR Therapists	Standard-of-Care Therapists	Analysis	*P* Value
No. of motivational techniques used per therapy session, median (IQR)	1.0 (0.5-1.9)	1.1 (0.05-2.5)	Mann-Whitney = 78.00	.94	24.4 (21.0-37.3)	2.3 (1.1-2.9)	Mann-Whitney = .00	<.001
Patient active time, mean (SD), %	41.7 (6.7)	38.3 (11.5)	*t*_24_ = 0.84	.41	52.5 (6.6)	41.3 (6.8)	*t*_24_ = 4.15	.001
Pittsburgh Rehabilitation Participation score, mean (SD)^b^	4.3 (0.5)	4.3 (0.4)	*t*_24_ = 0.30	.77	4.7 (0.3)	4.4 (0.4)	*t*_24_ = 1.55	.13
Duration of therapy sessions, mean (SD), min	Physical therapy	NA	NA		45.6 (13.4)	48.6 (15.9)	*t*_371_ = −1.92	.06
	Occupational therapy	NA	NA		37.7 (12.1)	39.9 (13.0)	*t*_352_= −1.68	.09

Abbreviations: EMR, enhanced medical rehabilitation; IQR, interquartile range; NA, not applicable.

^a^ After training in EMR, therapists carried out more motivational techniques and attained higher patient active time per therapy session. In contrast, standard-of-care therapists, who were not trained in EMR, showed no change in their techniques from pretraining.

^b^ Participation score range of 1 to 6, with 1 indicating no participation and 6 indicating excellent participation.

### Training and Supervision for EMR

Training and coaching were provided by PT and OT coaches who were study investigators (M.B. and P.B.) and included 5 structured group training sessions. Individual and group coaching sessions continued throughout the study to maintain treatment fidelity with EMR. Additional information on training techniques and fidelity testing has been published.^24,37^

### Randomization

Eligible participants were randomized to either EMR or standard-of-care therapy. The study statistician (M.Y.) generated the randomization sequence. Enrollment of participants and randomization to study conditions were conducted by research staff; randomization assignment was concealed until determination of participant eligibility by use of sequentially numbered sealed envelopes. Randomization was stratified by site and depressive symptoms (defined by a Montgomery-Äsberg Depression Rating Scale^38^ score ≥15 [indicating clinically significant depression]) and blocked within strata using random permuted block sizes of 2 and 4. Patients in the EMR arm received their weekday PT/OT only from EMR-trained therapists, while patients in the standard-of-care arm received PT/OT only from licensed therapists not trained in EMR. Otherwise, the 2 conditions did not differ (eg, EMR therapists were not instructed to spend more time with patients). To further equate the 2 conditions, the standard-of-care therapists also received 5 hours of training on various rehabilitation topics (eg, standardized assessments), and the therapists’ training was described neutrally to patients at the time of consent to avoid causing an expectancy bias.

### Measurement of Therapists’ Techniques in the Study

Research assistants observed study therapists and measured the following for both the EMR and standard-of-care groups: (1) therapists’ fidelity (adherence) to EMR^37^ (ie, whether the therapist carried out a correct technique at the appropriate time in therapy); (2) rehabilitation engagement of the patient with the therapist, using the Pittsburgh Rehabilitation Participation Scale,^39^ which is a 1-item Likert-type scale that assesses how actively the patient participates and engages in the therapy session, ranging from 1 (refused entire therapy session) to 6 (participated 100% and was actively engaged throughout therapy session); and (3) patient active time,^40^ which measures the percentage of time during the therapy session in which the patient actively performs a therapeutic activity (eg, walking or practicing an activity of daily living) as opposed to sitting and resting.

All of these techniques or processes were measured in a random sample of 5 therapy sessions per therapist before training to confirm that therapists’ skills were equivalent before training. A sample of 4 therapy sessions (2 OT and 2 PT) were then conducted for each randomized participant during their SNF stay to demonstrate that EMR active ingredients were delivered in a manner distinct from standard-of-care therapy (including demonstrating the absence of contamination of EMR into the standard-of-care arm).

### Outcome Measures

All outcomes were measured by blinded assessors. The primary outcome was change in Barthel Index score from admission to discharge. The Barthel Index is an instrument that measures a person’s ability to perform 10 basic activities of daily living or mobility items, with a scale range between 0 and 100 (higher scores indicate better function).^41^ It has proven external validity in estimating care needs and independent living in patients who have experienced stroke or hip fracture.^42,43^ The admission and discharge Barthel Index items were measured by 2 of us (M.B. and P.B.) from nursing and therapy notes that were redacted to hide participant identity or treatment assignment.

Secondary outcomes were self-reported function, performance-based gait measures, discharge to community settings, and rehospitalization. Self-reported function after discharge was assessed using a patient self-report version of the Barthel Index at days 30, 60, and 90 after randomization administered by telephone; we also ascertained whether rehospitalization occurred after completion of SNF rehabilitation up to day 90 after randomization. Performance-based measures obtained at admission and discharge from the facility were gait speed using 1 trial of the 10-m walk test^44,45^ and a 6-minute walk test (number of feet walked in 6 minutes).^46^ Gait assessments were videotaped and scored by a blinded assessor. Disposition from the SNF was dichotomized as return to the community setting (ie, to a private residence, group home, or assisted living) vs not returned (ie, to further skilled nursing rehabilitation, long-term care, or a hospital).

We collected 3 baseline variables for prespecified moderator analyses: the Montgomery-Äsberg Depression Rating Scale for evaluating the severity of depressive symptoms (range, 0-60; higher scores indicate more depressive symptoms; no cutoff was used in this study)^38^; the Short Blessed Test, a brief assessment of orientation, registration, and attention^36^ (higher scores indicate worse impairment, with scores of 5-9 consistent with mild cognitive impairment and ≥10 consistent with dementia); and the Cumulative Illness Rating Scale for Geriatrics score to quantify chronic illness burden from the medical records (higher scores indicate greater burden).^47^

### Statistical Analysis

The statistical analysis plan is available in Supplement 1. Intention-to-treat analysis was used. Study data were managed using REDCap, version 7.^48^ Analyses were performed using R, version 3.5.0 (R Foundation) or SPSS, version 24 (SPSS Inc). The primary outcome was change in Barthel Index score; the secondary outcomes were the 6-minute walk test and gait speed values at SNF discharge, as well as discharge disposition, rehospitalizations, and self-reported function at days 30, 60, and 90. The discharge point was the sole focus for the 6-minute walk test and gait speed test, as most participants were unable to complete these tasks at baseline and, therefore, there was no variability in these data at baseline. To test the primary hypothesis that EMR participants showed greater change in the Barthel Index score than standard-of-care participants, we used a marginal model with time (baseline and discharge), condition (EMR vs standard of care) and time × condition as fixed effects and with an unstructured covariance structure specified based on bayesian information criterion.

The secondary analyses for 6-minute walk test distance and gait speed used a Mann-Whitney test to examine differences in means between groups. For the secondary outcome of rehospitalization, χ^2^ analysis was used to determine whether rehospitalizations were dependent on condition. The outcome of self-reported function applied a marginal model using time (30, 60, and 90 days), condition, and time × condition as fixed effects, with the unstructured covariance structure specified based on the Bayesian information criterion. In addition, a marginal model tested for potential moderator effects: the model was constructed for each potential moderator and consisted of time and condition as factors, all 2-way interactions (eg, condition × time), and the 3-way interaction between time, condition, and the potential moderator, the latter of which is the term of interest. Exploratory analyses tested whether the effects of age, sex, race, and site altered the conclusions of the primary results as well as the conclusions of the moderator results. Both condition and site were constructed as 2-level fixed factors (EMR vs standard of care and facility 1 vs facility 2, respectively).

The trial was stopped at the end of the grant funding period (July 13, 2018). The study was originally powered to detect a moderate effect size of 0.4 based on a sample size of 252 with 80% power for 2 coprimary outcomes: functional change in the entire sample and change in depressive symptoms among participants who were clinically depressed as defined by a current depression diagnosis (major or minor depression, according to the Structural Clinical Interview for *Diagnostic and Statistical Manual of Medical Disorders-IV* Axis I Disorders^49^) at the time of SNF admission; however, of the 229 participants randomized, only 14 met a depression diagnosis, so this outcome analysis was not done and the level of significance, determined with 2-tailed testing, was set to 5% for functional change.

## Results

Of 3265 patients screened for study eligibility, 2909 were ineligible (eg, age <65 years, severe cognitive impairment, or not scheduled to receive ≥2 weeks of therapy), 127 refused to participate, and 229 were randomized (Figure). Table 2 reports the sample’s baseline characteristics; mean (SD) age was 79.3 (8.0) years and 149 were women (65.1%). The participants were ethnically diverse (177 [77.3%] white, 51 [22.3%] black, 1 [0.4%] >1 race); had a range of primary impairments along with multiple medical comorbidities, as measured by the Cumulative Illness Rating Scale for Geriatrics (mean [SD] score, 16.9 [5.2]); were highly disabled, as measured by the Barthel Index on admission (mean [SD] score, 33.5 [13.0]); and had a range of cognitive impairment symptoms, as shown on the Short Blessed Test (mean [SD] score, 4.1 [3.4]), and depressive symptoms, as shown on the Montgomery-Äsberg Depression Rating Scale (mean [SD] score, 8.6 [7.8]).

**Figure.**
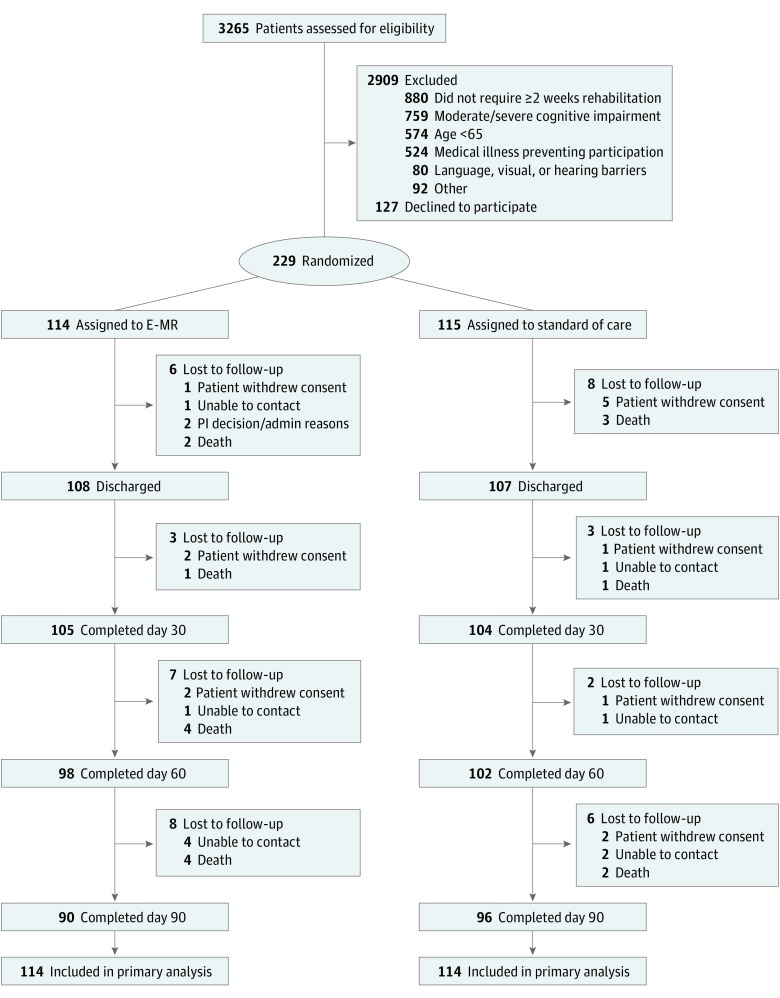
Participant Flow^a^ admin indicates administrative; EMR, enhanced medical rehabilitation; PI, principal investigator. ^a^Of the 115 patients assigned to standard of care, 1 participant withdrew before providing sufficient data for primary analysis.

### Process Data in Therapists Before and After Training

Before any training was conducted, the EMR therapists were similar to the standard-of-care therapists in terms of fidelity to the EMR intervention (quantified as number of engagement/motivational techniques consistent with EMR per therapy session), mean patient active time per therapy session, and patient engagement as measured by the Pittsburgh Rehabilitation Participation Scale (Table 3). After training (ie, during the randomized clinical trial), EMR therapists used a median (interquartile range) of 24.4 (21.0-37.3) motivational messages per therapy session compared with 2.3 (1.1-2.9) for nontrained therapists (*P* < .001). EMR patients were active during a mean (SD) of 52.5% (6.6%) of the therapy session time vs 41.3% (6.8%) for nontrained therapists (P = .001).(Table 3). Thus, therapists trained and coached in EMR were conducting this intervention with good fidelity that was clearly differentiated from standard of care, and these differences were attributable to the EMR training and coaching and not preexisting therapist differences. Table 3 also presents the mean (SD) durations of therapy sessions, showing that EMR sessions were not longer than standard-of-care sessions.

### Outcomes

Table 4 reports study outcomes in the EMR and standard-of-care groups. The main finding was that the EMR group had better functional recovery in terms of Barthel Index score change from admission to discharge—the study’s primary end point. A significant condition × time interaction was detected, with EMR participants exhibiting greater improvement during their SNF stay than standard-of-care participants. mean increase in Barthel Index score, 35 points (95% CI, 31.6-38.3 points) vs 28 points (95% CI, 25.2-31.7 points) (*P* = .007). There was no evidence of a difference in the length of stay (mean [SD], 23.5 [13.1] days). Adding age, sex, race, and site as main effects did not alter the conclusions. The eTable in Supplement 2 presents the individual item changes in the Barthel Index by group.

**Table 4.** Outcomes of Participants in the EMR and Standard-of-Care Groups^a^

Outcome	EMR	Standard of Care	Analysis	*P* Value
**Primary **				
Barthel Index, estimated marginal score, mean (SE)^b^	34.92 (1.66)	28.48 (1.68)	Time × condition: *F*_1220.59_ = 7.46; Cohen *d* = 0.36	.007
**Secondary**				
Gait speed on discharge, median (IQR), m/s	0.35 (0.47)	0.45 (0.49)	Mann-Whitney = 4292.50	.11
6-min walk on discharge, median (IQR), ft	170 (323)	210 (302)	Mann-Whitney = 4788.50	.91
Self-reported Barthel Index score at days 30, 60, and 90, mean (SE), estimated marginal^b^				
Day 30	78.79 (2.08)	78.95 (2.08)	Time: *F*_2148.06_ = 8.94	<.001
Day 60	84.27 (1.99)	85.01 (1.94)	Condition: *F*_1200.39_ = 0.06	.80
Day 90	83.65 (2.20)	84.67 (2.16)	Time × condition: *F*_2148.06_ = 0.04	.96
Discharge disposition to home vs institution, No. (%)	94/110 (85.5)	89/110 (80.9)	χ^2^_1_ = 0.81	.37
Rehospitalization, No. (%)	42/111 (37.8)	43/110 (39.0)	χ^2^_1_ = 0.04	.85
**Moderator Results (Barthel Index as Outcome)**				
Montgomery-Äsberg Depression Rating Scale^c^	NA	NA	Time × condition × test: *F*_1218.63_ = 0.01	.95
Cumulative Illness Rating Scale for Geriatrics^d^	NA	NA	Time × condition × test: *F*_1210.56_ = 0.06	.81
Short Blessed Test^e^	NA	NA	Time × condition × test: *F*_1218.36_ = 0.16	.69

Abbreviations: EMR, enhanced medical rehabilitation; IQR, interquartile range; NA, not applicable.

^a^ Patients randomized to EMR showed a greater improvement in the primary outcome (Barthel Index) from admission to discharge from the skilled nursing facility. There were no differences between conditions in any of the secondary outcomes, and there were no differences as a function of baseline depression, cognitive function, or level of medical comorbidities (moderator variables).

^b^ Scale range, 0 to 100, with higher scores indicating greater levels of function.

^c^ Scores of 15 or higher indicate major depression.

^d^ Higher scores indicate greater comorbid burden.

^e^ Scores of 5 to 9 consistent with mild cognitive impairment and 10 or higher consistent with dementia.

There was no evidence of a difference between EMR and standard-of-care participants in any of the secondary outcomes. Groups were not significantly different at discharge for gait speed (meters per second) and 6-minute walk test (meters). Self-reported function at days 30, 60, and 90 was not different between EMR and standard of care (mean [SE] Barthel Index score at day 90: EMR, 83.65 [2.20]; standard of care, 84.67 [2.16]; *P* = .96). In addition, there was not a main effect of condition; however, there was a significant main effect of time, showing that both groups reported improvement over time. Adding age, sex, race, and site as main effects did not alter the conclusions. Discharge disposition data (discharge to home vs institution) were available on 220 participants. Whether a participant was discharged to home or was institutionalized was independent of condition. Rehospitalization data during the 90-day follow-up were available on 221 participants. Whether a participant was rehospitalized was independent of condition. In terms of adverse events, there were none that were related to the study procedures or interventions.

### Moderator Results

We considered 3 variables measured on admission as potential moderators: depressive symptoms (Montgomery-Äsberg Depression Rating Scale score), level of cognitive impairment (Short Blessed Test score) and total amount of medical morbidity (Cumulative Illness Rating Scale for Geriatrics score). Results are presented in Table 4. Contrary to our hypothesis, none of these variables was a significant moderator of the effectiveness of EMR, as evident by a lack of a significant time × condition × moderator interaction. These results were not affected when including age, sex, race, and site as main effects in the models. For example, among those with low Montgomery-Äsberg Depression Rating Scale scores, participants randomized to EMR improved by a mean (SD) of 36.2 (17.4) points in the Barthel Index vs 28.3 (18.1) in the standard-of-care group; among those with high Montgomery-Äsberg Depression Rating Scale scores, the improvements were 32.7 (19.0) with EMR vs 28.7 (15.2) with standard-of-care. Similarly, improvements were 34.4 (18.4) with EMR vs 27.5 (17.1) vs standard-of-care with low (nonimpaired) Short Blessed Test scores, compared with 35.7 (17.4) with EMR vs 30.0 (17.5) with standard-of-care with high (impaired) Short Blessed Test scores.

## Discussion

This randomized clinical trial evaluated the effects of EMR, an approach to engage and motivate patients in PT/OT. Our main finding is that patients treated by EMR therapists had an estimated 25% greater functional recovery on average during postacute SNF rehabilitation, compared with those who received standard-of-care therapy. With respect to secondary outcomes, however, there were no group differences in gait measures, discharge disposition, or longer-term self-reported function. These findings are important because they indicate that older patients can achieve better short-term functional outcomes when treated by therapists who are trained and coached to systematically motivate patients and strive for higher-intensity therapy. Additional strategies are needed to maintain these functional gains after discharge from the rehabilitation facility and affect outcomes such as rehospitalization.

To our knowledge, this is the first full-scale test demonstrating benefits of a standardized method to improve rehabilitation outcomes by increasing engagement and intensity of therapy sessions. Functional recovery of older adults is an important outcome^50^ and one that is not always achieved despite postacute rehabilitation services.^51^ This finding fits well with the 2008 Institute of Medicine report *Retooling for an Aging America*, which recommended models of treatment that make older persons more active partners in their own care.^52^ This finding is also supported by smaller studies demonstrating the benefits of systematic interventions to increase engagement and intensity in rehabilitation settings.^53,54^

The EMR model does not ask therapists to do anything technically different in their practice, such as specific exercises or therapy protocols. Instead, it integrates communication techniques into therapy, increasing the focus on treatment engagement and intensity, thus providing more potent therapy without more treatment time. As such, EMR could be applied to any rehabilitation setting. This finding is important, because we found no differences in key secondary discharge outcomes, including disposition (frequency of returning home) or gait measures (gait speed and 6-minute walk test). Therefore, to fully optimize outcomes, it may be necessary to combine EMR with additional components, such as techniques to increase muscle strength and stamina to improve gait performance,^55^ and a postdischarge care component to reduce rehospitalization.^6,56^

In addition, there were no differences in longer-term self-reported function. Self-reported function may be a different construct than therapist-measured function; furthermore, self-reported function appeared to show a ceiling effect by 60 to 90 days after SNF admission, suggesting that self-report scores were inflated or that participants who were able to be assessed after discharge were also those who regained most or all of their function. Studies should measure both self-reported and observed function to better understand the long-term functional trajectory after rehabilitation.

The effectiveness of EMR was not moderated by baseline levels of depression, cognitive impairment, or medical complexity, which we had estimated would be potential barriers to motivation and recovery. This finding argues against providing EMR only to certain patient groups, such as individuals with depression, in favor of a more universal application of this approach in rehabilitation.

### Strengths and Limitations

Strengths of this study included demonstrating that EMR and standard-of-care therapists were equated at baseline prior to training and blinding of outcomes. The study has several limitations. The trial was conducted in 2 facilities in 1 geographic area. Because of logistical challenges, we were unable to control therapy done on weekends and we were only able to assign and randomize participants after 1 to 2 days in the SNF. However, this limitation would not invalidate positive study findings; possibly, EMR would have greater effects if implemented for all therapy sessions. Further studies of EMR are needed to replicate and extend these findings. Another limitation is that, for feasibility reasons, we were unable to directly observe all therapy sessions and we did not have a method of assessing treatment fidelity by therapists other than by direct observation; this lack of continuous assessment could have influenced EMR-trained therapists to carry out more motivational messaging when observed. Other limitations include low statistical power for examining binary secondary outcomes, such as rehospitalization, and a high rate of SNF admissions excluded for reasons such as patients being severely cognitively impaired or not requiring intensive rehabilitation.

## Conclusions

This trial’s findings suggest that EMR is effective in improving functional recovery for older adults in postacute rehabilitation. Improving outcomes is paramount for the estimated 6.4 million older adults receiving rehabilitation services yearly,^51^ and the medical rehabilitation field has urged a greater focus on patient engagement and intensity in medical rehabilitation.^57,58,59^

## Notes

Supplement 1. **Trial Protocol and Statistical Analysis Plan**

Supplement 2. **eTable.** Barthel Changes by Individual Item in the Enhanced Medical Rehabilitation (EMR) and Standard of Care Groups

Supplement 3. **Data Sharing Statement**
