# Supplementary material for: Effect of Enhanced Medical Rehabilitation on Functional Recovery in Older Adults Receiving Skilled Nursing Care After Acute Rehabilitation: A Randomized Clinical Trial
Source: JAMA Netw Open. 2019 Jul 31;2(7):e198199. doi: 10.1001/jamanetworkopen.2019.8199 (PMC6669784; doi:10.1001/jamanetworkopen.2019.8199)
Supplement: Supplement 1. — Trial Protocol and Statistical Analysis Plan [file jamanetwopen-2-e198199-s001.pdf]

Version: 8
Submitted to IRB: 12/12/17

Enhanced Medical Rehabilitation R01 Study Protocol

Principal Investigator: Eric Lenze, MD

Co investigators:

Marghuretta Bland, PT, DPT, NCS, MSCI

Rebecca Birkenmeier, OTD, OTR/L

Ellen Binder, MD

Catherine Lang, PhD

Carolyn Baum, PhD

Nancy Morrow-Howell, PhD

Tom Rodebaugh, PhD

Peggy Barco, OTD, OTR/L, SCDCM

Study team members:

Emily Lenard, BA

Julie Schweiger, CCRC

Selmi Kallmi, BA
Sarah Pennock
Henry Mitchell
Gabriela Gonzalez
Dave Dixon, PhD

Michael Yingling, MS

Healthy Mind Lab Staff:
Michelle Voegtle, BA

Angela Stevens, BS

Marissa Rhea, MA
Brian Abboud

Andes Perez

Hayley Hinton, BA

Jissell Torres

Kelly Ahern

Supported by:

The National Institute of Mental Health (NIMH)

R01 MH099011

TABLE OF CONTENTS

ENHANCED MEDICAL REHABILITATION R01 STUDY PROTOCOL 1

A INTRODUCTION 3

A1 STUDY ABSTRACT 3
A2 STUDY AIMS AND HYPOTHESES 3
A3 PURPOSE OF THE STUDY PROTOCOL 4

B BACKGROUND 4

B1 PILOT RCT FINDINGS 4
B2 RATIONALE FOR THIS STUDY 5

C STUDY OBJECTIVES 7

C1 PRIMARY AIM 7
C2 SECONDARY AIM 7
C3 RATIONALE FOR THE SELECTION OF OUTCOME MEASURES 7

D STUDY INTERVENTION: ENHANCED MEDICAL REHABILITATION 8

E STUDY DESIGN 11

E1 DESIGN SUMMARY 11
E2 SUBJECT SELECTION AND WITHDRAWAL 11

F STUDY PROCEDURES 15

F1 SCREENING FOR ELIGIBILITY 15
F2 SCHEDULE OF MEASUREMENTS 15
F3 SNF ADMIT (BASELINE) VISIT 16
F4 DAY 30 VISIT 16
F5 SNF DISCHARGE 16
F6 DAY 60 VISIT 16
F7 DAY 90 VISIT 17
F8 DATA AND SAFETY MONITORING PLAN 17

G STATISTICAL PLAN 19

G1 SAMPLE SIZE DETERMINATION AND POWER 19
G2 INTERIM MONITORING AND EARLY STOPPING 20
G3 ANALYSIS PLAN 20
G4 STATISTICAL METHODS 20

H DATA HANDLING AND RECORD KEEPING 21

H1 CONFIDENTIALITY AND SECURITY 21
H2 TRAINING 21
H3 RECORDS RETENTION 21

I STUDY MONITORING, AUDITING, AND INSPECTING 22

J STUDY ADMINISTRATION 22

J1 ORGANIZATION AND PARTICIPATING CENTERS 22
J2 FUNDING SOURCE AND CONFLICTS OF INTEREST 22
J3 PARTICIPANT PAYMENT 22

K PUBLICATION PLAN 22

L REFERENCES 23

A Introduction

A1 Study Abstract

For millions of disabled older adults each year, post-acute rehabilitation in skilled nursing facilities (SNFs) is a narrow window of opportunity to regain enough function to return home and live independently -- but all too often they fail due to problems such as depression that undermine rehabilitation's benefits.

Therefore, we created Enhanced Medical Rehabilitation (EMR) for older adults. A real-world adaptation of the science of behavior change, EMR is an integrated set of skills for physical and occupational therapists (PT/OT) that transform standard post-acute rehabilitation through: (1) a patient-directed, interactive approach (2) increased rehabilitation intensity (3) frequent feedback to patients on effort and progress. We developed training and supervision techniques so that PT/OTs carry out these skills with high treatment integrity.

In NIMH R34 pilot work, we found: (1) EMR is high-intensity and engaging: therapy by PT/OTs trained and supervised in EMR is greatly amplified in intensity and patient engagement compared to standard-of-care rehabilitation. (2) EMR improves functional and affective recovery: patients randomized to EMR had better improvement of function, physical performance, and positive and negative affect. (3) EMR overcomes barriers to rehabilitation: patients with depressive symptoms, cognitive impairment, and multiple medical comorbidities benefitted most from EMR relative to standard therapy.

We propose a randomized trial that will test EMR's benefits over standard-of-care rehabilitation for affective and functional recovery. Our aims are (1) examine the effectiveness of EMR for improving functional and affective outcomes in 252 older adults admitted to SNFs for post-acute rehabilitation, and (2) examine EMR's ability to overcome patient-level barriers (such as depression) to successful rehabilitation.

The innovation of this project is high: it is a novel application of theories of behavior change to improve both affect and function. It responds to NIMH strategic plan objective 3, "Develop new and better interventions for mental disorders that incorporate the diverse needs and circumstances of people with mental illness."

The public health significance is also high: recovery from disablement is a health issue of enormous human and economic significance. Success in this line of research will make rehabilitation more effective. Doing so would benefit all older adults in this sector of care but particularly those with depression, cognitive impairment, and other complications. This project addresses NIMH's goal of interventions to improve mental health and related health outcomes in real-world settings.

A2 Study Aims and Hypotheses

Aim 1: Examine the effectiveness of EMR for improving functional and affective outcomes in older adults admitted to SNFs for post-acute rehabilitation.
H1: EMR will improve functional and affective recovery to a greater extent than standard-of-care rehabilitation.
Rationale: This test could demonstrate EMR's effectiveness, a key step toward establishing EMR as the gold-standard practice for post-acute rehabilitation, to the benefit of millions of older adults.

Aim 2: Examine EMR's ability to overcome patient-level barriers to successful rehabilitation.
H2: *The effectiveness of EMR for functional recovery will be greatest in: (a) patients with clinical depression; (b) patients with high levels of medical comorbidity; (c) patients with cognitive impairment.*
Rationale: Demonstrating that EMR is particularly effective in the most vulnerable patients could overturn conventional wisdom that such persons should be excluded from intensive rehabilitation.

A3 Purpose of the Study Protocol

This study protocol will serve as the working document for the study team to ensure the conduct of the research is consistent with the IRB approval.

B Background

Disabling medical events frequently occur in elderly persons; one study found that more than one-half of community-living elderly persons had a disabling medical event over a median four year follow-up (Hardy et al, 2004). In 2001 there were over six million hospitalizations in the US for persons aged 65 and older for disabling events such as heart disease, stroke, fracture, and osteoarthritis (National Hospital Discharge Survey, 2001), a number that will dramatically increase in the next 20-30 years as the number of persons 65 and older increases from 35 million (in 2000) to more than 86 million by 2050 (US Bureau of the Census, 2004).

B1 Pilot RCT Findings

From 2010-2011 we carried out an NIMH R34 treatment development grant to develop and preliminarily test Enhanced Medical Rehabilitation, a novel PT and OT intervention. Our goal was to apply the science of behavior change to overcome shortcomings of standard of care rehabilitation, namely that it does not provide high-intensity and highly engaging therapy.

We trained and supervised four therapists (2 OT, 2 PT) and we monitored their fidelity to the Enhanced Medical Rehabilitation model. In a case series, we found excellent functional recovery and reduction of depressive symptoms with Enhanced Medical Rehabilitation. Then, we carried out a pilot RCT, randomizing 26 participants to Enhanced Medical Rehabilitation by the trained therapists or standard of care rehabilitation by non-trained therapists.

In this pilot RCT we found the mechanism of Enhanced Medical Rehabilitation is increased engagement and increased intensity in therapy sessions; both constructs are considered critical in the rehabilitation sector, yet surprisingly little research has advanced their measurement. Enhanced Medical Rehabilitation sessions markedly outpaced standard-of-care sessions (by 2-3 fold) in measures of therapy intensity. Enhanced Medical Rehabilitation participation scores were higher also in patient engagement, indicating more active engagement by patients. In contrast, scores from the Working Alliance Inventory (WAI) are similar (and high) in both Enhanced Medical Rehabilitation and SOC. The WAI examines non-specific therapist variables and may therefore be a technique-independent predictor of positive rehabilitation outcomes. Thus,

these data indicate that the differences seen are specifically related to higher therapy engagement in Enhanced Medical Rehabilitation, not non-specific factors (such as liking the therapist).Additionally, among those with high baseline depressive symptoms, Enhanced Medical Rehabilitation resulted in greater improvement in negative affect: a mean reduction of 5.6 points, vs 2.2 points in SOC rehabilitation (scale's range was 8-40). The relative benefits of Enhanced Medical Rehabilitation may be greatest in patients with depression, cognitive impairment, and multiple medical comorbidities. We developed Enhanced Medical Rehabilitation because these common comorbidities often undermine successful rehabilitation, likely via demoralization or lack of insight. When we dichotomized the sample based on present/absent cognitive impairment, high/low medical complexity (number of medical conditions), or high/low depressive symptoms at baseline, in each case we found trends for greater relative recovery in Enhanced Medical Rehabilitation compared to SOC among those with higher impairments. This appeared to be because SOC did not work as well (lower functional recovery in those with these comorbidities) while Enhanced Medical Rehabilitation did work as well (similar functional recovery with or without these comorbidities. These results, while very preliminary, suggest that Enhanced Medical Rehabilitation does overcome barriers to successful rehabilitation, which is consistent with its theoretical focus on overcoming barriers to engagement.

B2 Rationale for this Study

2.a The growing role of post-acute rehabilitation

Americans are aging and having more medical events such as heart attack, stroke, and hip fracture.[1] More than ever, older adults are surviving these events and leaving the hospital, alive but severely disabled.[2-4] The solution for these highly disabled older adults is rehabilitation in the post-acute care setting, a large and rapidly-growing sector of care.[5] Post-acute rehabilitation consists of daily physical therapy (PT) and occupational therapy (OT) to achieve restoration (e.g., regain walking ability and counteract bone and muscle loss by gait and muscle strengthening exercises), and adaptation (e.g., learn to perform activities of daily living and safely live at home).

The most common setting for post-acute rehabilitation is a skilled nursing facility (SNF). Length of stay is about 3 weeks, a narrow window in which to recover and return home or be institutionalized.

2.b Depression is the most common and deleterious mental disorder in post-acute rehabilitation.

Disabled older adults have high rates of depressive symptoms and disorders which lead to and amplify disability. This bidirectional relationship between late-life depression and disability is intensified in post-acute rehabilitation, where affective impairments are barriers to successful rehabilitation.[6,7] In other words, in this population, depressive symptoms are both an outcome of rehabilitation (as disability is depressogenic) and moderate (reduce) rehabilitation success. Additionally, cognitive impairment and high levels of medical complexity impede rehabilitation success.

Existing mental health interventions are a poor fit for these disabled and depressed older adults.[8-10] Post-acute rehabilitation is a difficult setting in which to integrate evidence-based mental health treatments, due to short stays, difficulty of case detection, and

competing demands (most notably from the rehabilitation itself). Additionally, frail and medically ill older adults have less benefit and greater risks from antidepressants.[11] Finally, the affective state of these older adults appears tightly linked to the disablement itself.[12-16] Therefore, an optimal intervention would use a common conceptual model to address affective impairments together with the disablement. Trans-NIH initiatives such as the NIH Integrated Health Improvement Strategies Workgroup[17] have called for more research in such treatment models.

2.c Do we need to enhance rehabilitation?

Post-acute PT/OT itself could be this optimal intervention, ameliorating affective impairments and disablement via intensive goal-oriented interactions with highly-trained providers. Yet two factors have been observed to undermine rehabilitation, particularly for depressed older adults: (1) The PT and OT is of low intensity, often too low to provide adequate restoration of function and improve affect;[18,19] (2) The interaction with patients is unengaging[20] because (a) therapists are directive, telling patients what to do, and (b) therapists do not provide adequate feedback on patients' effort and progress in therapy, such that patients will know where they stand in their recovery and why their therapy activities are important.

From a behavior change standpoint, standard rehabilitation fails because it is a traditional Action-oriented program, assuming that patients are motivated to carry out therapeutic activities and not demoralized or uninsightful.[21] Yet our research in post-acute rehabilitation shows that a substantial proportion are not adequately motivated and do not participate well in their therapy[22], particularly when they have depressive symptoms and/or cognitive impairment.[23,24] When participation in therapy is poorer, recovery is poorer.[6,23-29]

The conventional wisdom in rehabilitation practice guidelines is a distortion of this observation; it states that inadequately motivated patients are "poor candidates" for high-intensity rehabilitation.[30,31] This conventional wisdom not only ignores behavior change research, it has no evidence basis; indeed, it runs counter to our observation that depressed and apathetic older adults do better in high-intensity than lower-intensity rehabilitation.[27] Yet, no research has clearly refuted this wisdom. The resultant disparity is that older adults most at risk for poor outcomes after disablement – such as those with depression or cognitive impairment – may be relegated to the least intense rehabilitation.[32]

To summarize the significance, post-acute rehabilitation is low-intensity, low-engagement and too often fails to achieve functional recovery, especially in the most vulnerable individuals. This problem should be remediable by applying the science of behavior change to this setting. We therefore developed Enhanced Medical Rehabilitation.

C Study Objectives

C1 Primary Aim

Aim 1: Examine the effectiveness of EMR for improving functional and affective outcomes.
H1: EMR will improve functional and affective recovery to a greater extent than standard-of-care rehabilitation.

Explanation of H1: The purpose of H1 is to examine the effectiveness of EMR. If H1 is supported, this could establish EMR as the gold-standard practice for post-acute rehabilitation. Our primary analysis is the change from baseline to Day 30. This single primary endpoint allows for an effectiveness test that is not confounded by length of stay differences or post-discharge issues beyond the reach of EMR.

C2 Secondary Aim

Aim 2: Examine EMR's ability to overcome patient-level barriers to successful rehabilitation.
H2: The effectiveness of EMR for functional recovery will be greatest in: (a) patients with clinical depression; (b) patients with high levels of medical comorbidity; (c) patients with cognitive impairment.

Explanation of H2: EMR was designed to overcome barriers to rehabilitation (for example, due to depression); therefore, the difference in functional recovery between EMR and SOC should be greater in the most vulnerable older adults. If H2 is supported, we will demonstrate that older adults with these common comorbidities benefit the most from high intensity, high engagement rehabilitation. If so, this would refute conventional wisdom in post-acute rehabilitation, codified in admission criteria for rehabilitation facilities, regarding the appropriate candidates for rehabilitation (as described in Significance).[32]

C3 Rationale for the Selection of Outcome Measures

Consistent with the common conceptual model of EMR improving affective and functional recovery, this project has two co-primary outcomes, one of functional recovery, and one of affective recovery. Our primary measure of **functional outcome** is the **Barthel Index**, a 0-100 scale[63] that measures ability (time and physical assistance required) to perform 10 basic Activities of Daily Living or mobility items making it optimal for this highly debilitated population.[62,64-66] The Barthel Index has excellent external validity: a higher score predicts greater likelihood of being able to live at home and degree of independence following discharge from the hospital.[67,68] Secondary measures are gait speed (time to walk 6 meters, in meters/second) and 6 minute walk (number of feet walked in 6 minutes). These performance-based measures of physical function have found increasing favor as outcome measures in rehabilitation intervention studies, because of their strong external validity in predicting clinical outcomes such as hospitalization and mortality.[3,69-71]

*The primary measure of **change in depression** is the Montgomery Asberg Depression*

Rating Scale, a commonly-used depression measure in clinical trials. We have used it in many prior studies of older adults. Our secondary measure is an 18-item positive and negative affect scale that measures positive and negative (depressed and anxious) affect. It measures the dominant dimensions of human emotional experience, which have important roles in both quality of life and as a predictor of success in geriatric rehabilitation. It is a combination of the full 10-item positive affect scale of the Positive and Negative Affect Schedule (PANAS),[77] plus five items from the brief PANAS measuring negative affect[78] and the three non-reverse coded items from a brief State Anxiety Scale[79] derived from the State Trait Anxiety Inventory to measure negative (anxious) affect. This scale uses participants' responses to questions (e.g., "how anxious do you feel at present") to assess their current feeling or affective states. The PANAS has excellent reliability in post-acute rehabilitation, where it is considered valuable in capturing the range of mood and motivational issues in this setting.[80] Our addition of specific anxiety items reflects the high rate of anxiety problems in post-acute rehabilitation.[29,81-84] Additionally, this dimensional assessment of affect responds to calls by NIMH to use as outcomes dimensions that reflect more basic, validated emotional or behavioral states, as reflected in the Research Domain Criteria.[85] These depression/affect measures will only be carried out in participants with a baseline clinical diagnosis of current major or minor depressive episode, based on the Structured Clinical Interview for DSM-IV (SCID)[86] which we will carry out at baseline prior to randomization. We have considerable experience with the SCID in this population from prior NIMH-funded studies. Based on our pilot RCT experience, about 25% of eligible participants will have a current diagnosis; we will enrich the sample by continuing to recruit to ensure that 50% of randomized participants have a depression diagnosis, so that this study is adequately informative regarding benefits for late-life depression in this setting. Our test-retest Kappa (using different, blinded raters) for major/minor depression in this setting is 0.8 (good).

The measures we have chosen have adequate psychometric characteristics in this setting and are minimally burdensome. We will monitor all of the assessment procedures closely during the RCT (inter-rater and test-retest), and if reliability of any measure falls below ICC=.8 (or Kappa 0.7) we will re-train research staff.

D Study Intervention: Enhanced Medical Rehabilitation

EMR is a set of skills for PT and OT to increase both intensity and the engagement of all therapeutic sessions. It has three foci for therapy sessions: high intensity ("optimizing intensity"), feedback to patients on their effort and progress ("linking activities to goals"), and a patient-directed approach ("patient as boss"). EMR is a "how" intervention, not a "what" intervention: its skill set integrates into existing OT/PT, rather than adding new activities or exercises or adding another specialist to the setting. In other words, OT or PT in the EMR model is still OT or PT (which in this setting is individualized by the therapist to the patient's impairments, abilities, home environment, and other contextual factors). The difference is in the effort to engage the patient and provide high-intensity therapy. For this reason, EMR can integrate well into post-acute OT/PT no matter what the patient's primary impairment, comorbidities (cognitive, medical), or other contextual factors.

EMR's foci have deep roots in the science of behavior change, including social cognitive theory,[33] self-regulatory theory,[34] the Theory of Planned Behavior,[35] and the Transtheoretical Model which encompasses these and other theories.[21] **EMR's three**

foci are described with an explanation of their innovation for rehabilitation and their link to theories of behavior change here:

1. Interactive, patient-directed approach

a. In the first session, OT and PT elicit the patient's own goals for rehabilitation using the Rehabilitation Goals Interview, a brief (15 minute) semi-structured interview to generate patient-nominated therapy goals.[36] Up to 5 goals are nominated by the patient as most important to them. We found this technique to provide more rich and precise information than the standard rehabilitation technique of simply asking patients what their goals are. This interview is linked to the Transtheoretical (Stages of Change) Model in that it raises the patient's awareness of themselves presently in contrast to where they want to be. The interview frequently produces an emotional experience by the patient, and so therapists are also trained to expect this and work with it. Regarding innovation: no rehabilitation therapists, to our knowledge, use a structured interview to discern patients' goals (they typically ask "what are your goals?" which is generic and insufficient).

b. These patient-reported goals become the basis for all interactions:

   i. "You decided that helping care for your grandchildren, getting to church, and walking your dog are your goals. Which one of those would you like to focus on today?"

   ii. "Okay, so we'll focus today's therapy on activities that will get you closer to your goal of being able to help take care of your grandchildren again. What will you need to be able to do so that you can get back to doing that?"

   iii. "Just to make sure we're on the same page, can you talk me through how this activity will get you closer to your goal of helping care for your grandchildren?"

   iv. Additionally, study staff (not blinded raters) make a videotape of the patient's home, which the therapists watch alone and again with the patient. This helps to further link (for both patient and therapist) rehabilitation activities with goals related to functioning at home safely.

   Linking actions to patient goals helps move patients into an action phase, in accordance with the Transtheoretical Model; similarly it increases intention according to the Theory of Planned Behavior. This explicit linking of therapy activities with the patient's goals is novel; it is not done repeatedly and purposefully by any rehabilitation therapist, in our experience.

c. To choose therapy activities, therapists "ask, don't tell": "What activity would you like to do next?" Non-directive interaction is consistent with Social Cognitive Therapy (increasing self-control), Self-Determination Theory, and the Transtheoretical Model (self-liberation). In post-pilot study interviews, the real-world therapists told us that this skill was one of the more novel (i.e., not part of their previous training or practice) and important features of EMR.

d. Along the same lines, therapists check in with the patient after each activity or exercise: "How do you feel you did with those stairs?"…"It seems you weren't happy with how you did; how can you make getting from the bed to the wheelchair easier or safer?"

e. These interaction strategies are also used at a therapist meeting with the patient and caregiver(s).

f. Therapists are trained and supervised to use open-ended questions when appropriate, to avoid directive language in general, and to avoid the use of jargon.

Points d-f help EMR therapists to increase their patient's sense of self-control and perceived behavioral control, as well as maintain patient investment, and increase rapport.

2. **Increased intensity**
    a. Therapist guides patient towards higher-intensity activities:
        i. "Let's start off with a challenging activity. Which one of those activities we just discussed would you like to try first?"
        ii. "How hard are you working?" (patient responds "4" on 1-10 scale, indicating that therapeutic exercise requires little effort) "We'd like to get you even stronger. What would it take to get you up to a 7 or 8 while doing this activity?"
    b. Exercises and activities are individualized to maximize effort ("Do you want to see how much farther down the hall you can walk?"), not of arbitrary length ("now walk 10 feet").
    c. Therapists are trained and supervised to minimize down-time during therapy sessions.

    The focus on maximizing intensity is both novel and an advance over standard therapy in which the level of intensity is either arbitrary or at most is encouraged non-specifically. Increased intensity is also closely linked to the other EMR foci and their behavioral theory underpinnings. The 1-10 perceived effort scale, and feedback ensuing from it, is an innovation of EMR for rehabilitation settings. It is based on a scale developed for community-dwelling elders relative to exercise prescription.[37]

3. **Frequent feedback on effort and progress**
    a. Therapist tells patient the benefits when therapeutic activity/exercise was hard: "How hard is this exercise?" (patient responds "9", indicating that therapeutic exercise was very difficult) "I can see you are working hard, and your heart is beating fast. That means <u>right now</u> you are increasing your stamina…your endurance and your heart and lung capacity are getting better."
    b. Therapist comments on progress when an activity becomes easier: "You rated your effort a 3 on this stair-climbing, and last week you rated it an 8. Can you see that you are getting stronger and closer to your goals?"
    c. Therapist links patient's progress in activities to goal achievement: "Remember when you told me that you wanted to be able to walk your dog again? Well, today, you were able to walk 15 feet without much assistance. You're closer to your goal."
    d. Therapists use a progress binder to systematically review progress with patients in all activities, each Monday, Wednesday, and Friday after therapy, and link it to goal attainment.

    a-d above are consistent with the feedback focus of self-regulatory theory and also with social cognitive theory and the Transtheoretical Model in that they are intended to increase self-efficacy (or perceived behavioral control) and improve outcome expectations.

e. Therapists are trained to understand and manage displays of affect by the patient. If a patient shows emotional distress, the therapist shows empathy, acknowledging the affect prior to continuing therapy. If a patient appears to the therapist as amotivated or disinterested, the therapist conceptualizes this as a temporary state (rather than an immutable trait) and tries to find a solution with the patient.

f. Therapists are supervised regarding communication skills such as eye contact, speaking at the level of the patient, and avoiding jargon.

e-f are critical interaction skills to improve rapport-building, and they underlie all other aspects of EMR. They require de novo training (in the case of managing affect) or retraining (in the case of basic communication skills because they are not reinforced to therapists in real world settings).

E Study Design

E1 Design Summary

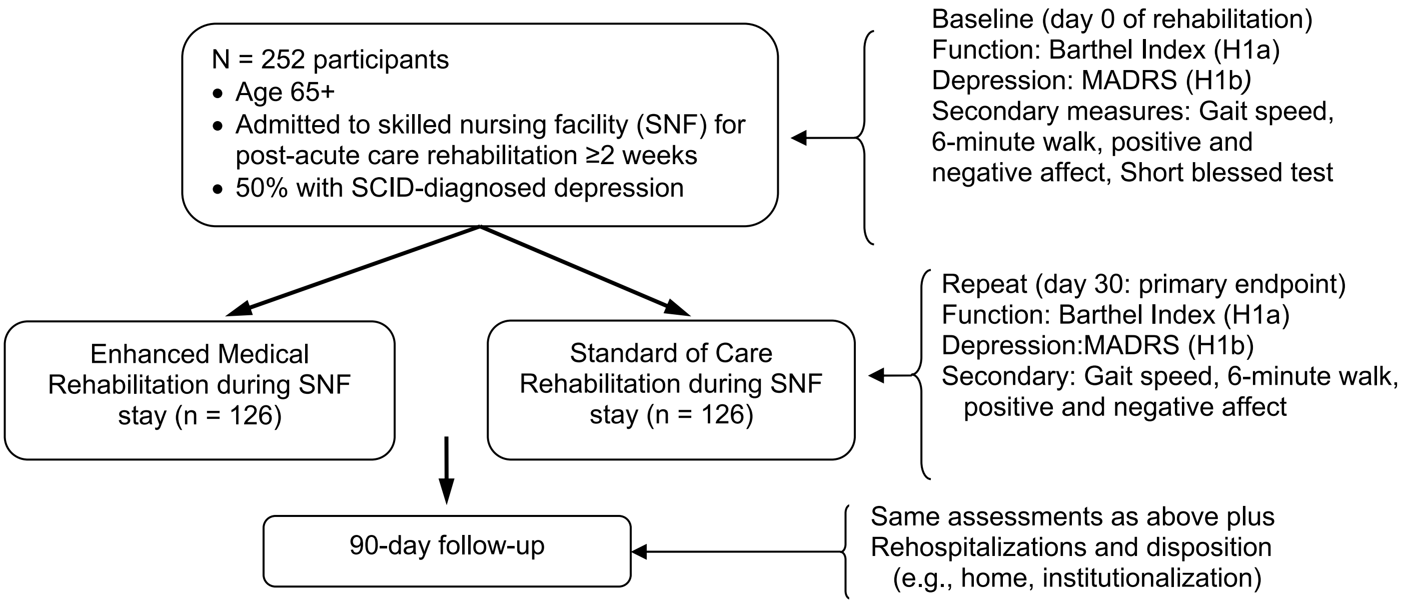

E2 Subject Selection and Withdrawal

2.a Inclusion Criteria

Participants must be 65 and older, and be admitted to a skilled nursing facility for post-acute care from PT and OT for 2 weeks or more.

2.a Exclusion Criteria

- Language, visual or hearing barriers to participation (e.g. unable to communicate with research staff).

- Medical illness preventing study participation or accurate data collection (e.g., highly unstable cardiac illness such that early re-hospitalization is expected; metastatic or other cancer such that hospice is recommended or survival is limited;
- Moderate-severe dementia (demonstrated by chart diagnosis and/or short blessed score greater than 13);
- Progressive neurological condition such that recovery of function is not feasible;
- Patient did not have the ability to walk prior to hospitalization (e.g. paraplegic);
- Schizophrenia or other chronic or current psychotic disorder.
- Patient normally resides in a long term care facility.

2.b Ethical Considerations

This study will be conducted in accordance with modern ethical standards for biomedical research, particularly in regards to avoiding conflicts of interest, informed consent, and protecting patient privacy.

2.c Subject Recruitment Plans and Consent Process

The study population consists entirely of patients at skilled nursing facilities and recruitment will be facilitated by our ongoing relationship with them. The recruitment and consent processes described below were developed with our original partner site, Barnes-Jewish Extended Care. Subsequent modifications may be needed to adapt this process to the needs of additional sites.

Clinical skilled nursing facility staff will alert the research team of an anticipated admission. The research team will review the patient's records at the skilled nursing facility to ensure inclusion/exclusion criteria.

After checking in with facility patient-care staff to confirm the availability of the potential participant, patients who appear appropriate per chart will be approached by WU research staff and told that they may be eligible for the enhanced rehabilitation study. To ensure participants' comfort, the study team will assess the setting and ensure that the individual is positioned such that they can comfortably complete any assessments, including writing in a reasonable position, that they have had an opportunity to go to the toilet, eat and are dressed (if appropriate). Just asking the participant may not be the best approach for all individuals as some may agree without actually feeling this it is the best time or situation. It is important that participants not put off their own needs (whether it be repositioning themselves, using the bathroom or getting dressed) and that the research assessments not interfere with this.

If the patient gives their assent, research staff will screen them for cognitive impairment, with the Short Blessed Test (SBT). If they do not assent to the screening then we will not ask them the questions and will not contact them further.

Patients who score 13 or higher will be assumed unable to provide consent and thus will be excluded from the study. Study staff and/or PI will use clinical judgment to determine whether to include patients who score between 10 and 12 on the SBT. If they are not cognitively impaired (score 0-9 on SBT) at the time of this screening we will go through the full informed consent process for the study.

Research staff will review the consent form with the patient in their room at the skilled nursing facility, encouraging the patient to ask questions throughout. The assessment of understanding tool will be used as needed to ensure each patient understands the procedures, risks, and benefits of the study.

2.d Randomization Method and Blinding

We will randomize 1:1 to EMR (i.e., all OT/PT sessions done by EMR-trained and supervised therapists) or SOC (i.e., all sessions done by non-EMR-trained therapists), as in our pilot RCT. We will stratify by SNF site and by baseline depression status. To prevent subversion of randomization,[61] the statistician will hold the randomization list, releasing an assignment once a participant is consented and eligible.

EMR group: The EMR group will be the same as in our pilot study; participants who are randomized to the EMR group will receive therapy only from therapists who are trained and supervised in EMR. Their therapy sessions will follow the EMR protocol. Otherwise their SNF care will not differ from usual care.
SOC group: The standard-of-care (SOC) group will receive therapy only from PT/OTs (or assistants) who are not trained nor supervised in EMR. These therapists are monitored (videotaped or observed) but not asked to do anything differently with their patients.

Blinding: Ratings will be conducted by a research associate who is blind to the study intervention, purpose, and randomization status. Patients will not be blinded to their randomization status, but will not be made knowledgeable about how exactly Enhanced Rehab is different from SOC. Therapists providing SOC treatment will be blinded to the principles of Enhanced Rehab to prevent contamination.

Open Label participants: Up to 12 participants at each site will be recruited as "open label pilot" participants. These participants will meet the same eligibility criteria as the randomized participants but will be automatically assigned to the Enhanced group. This will be done prior to beginning to randomize participants to ensure the quality of the intervention and data collection procedures.

2.e Risks and Benefits

Likely risks: There are no likely risks.

Less Likely risks: The enhanced rehabilitation intervention and the measures impose some risk of emotional discomfort. The enhanced rehabilitation may cause muscle soreness and fatigue. If patients are allowed to take Tylenol or NSAID they may do so with approval from their skilled nursing home doctor.

Rare risks: There is a potential breach of confidentiality with research records and with videotaping. There is a possibility of falling and fracture during the tests of your walking and balance. This will be minimized through the use of a safety belt during all testing procedures

Benefits: Research assessments may reveal conditions that had not been identified (e.g., depression), leading to management of the patients' depression or functional recovery that would not have occurred otherwise. Research intervention (i.e., Enhanced

Rehab) may lead to improvements in physical functioning (compared to standard of care).
The results of this research may benefit society in that it will lead to an intervention which addresses a major gap in medical/functional rehabilitation in an acute rehab setting.

2.f Early Withdrawal of Participants

Participants may be withdrawn prior to study completion for a variety of reasons. Reasons for withdrawal will be noted on a termination form. There are no risks associated with early withdrawal. Common reasons for early withdrawal include:

-Screen failures: (note which exclusion criteria was met)
-Participant withdraws from study: (note the participant's stated reason for withdrawing)
-Participant no longer able to comply with protocol: (note reason)
-Death: (note date of death and source of information)

2.g Data Collection and Follow-up for Withdrawn Participants

During a situation where a participant is unable to be interviewed due to incapacitation or death of participant, research staff will attempt to collect final prognosis information from participant's medical chart or by asking a family member.

Research staff will collect no further data from screen failures. Withdrawn participants will not otherwise be followed up with.

F Study Procedures

F1 Screening for Eligibility

Upon receiving notification of a newly admitted patient to Barnes-Jewish Extended Care, research staff will review patient for information related to eligibility criteria. If the patient appears to be eligible, they will be screened using the Short Blessed Test (see 2.c.).

F2 Schedule of Measurements

Prior to meeting with a participant to perform study assessments, study staff will check-in with facility patient-care staff to confirm the availability of the participant. To ensure participants' comfort, the study team will assess the setting and ensure that the individual is positioned such that they can comfortably complete any assessments, including writing in a reasonable position, that they have had an opportunity to go to the toilet, eat and are dressed (if appropriate). Just asking the participant may not be the best approach for all individuals as some may agree without actually feeling this it is the best time or situation. It is important that participants not put off their own needs (whether it be repositioning themselves, using the bathroom or getting dressed) and that the research assessments not interfere with this.

	**Schedule of Assessments**	**SNF admit (baseline)**	**Day 7**	**Day 30 (primary endpoint)**	**SNF discharge**	**Day 60**	**Day 90**
**Outcomes**	*Function*						
	**Barthel Index (primary outcome)**	X		X	X	X	X
	Gait speed	X		X	X		
	6-minute walk	X			X		
	Depression SCID (given if indicated by MADRS)	X					
	**MADRS**	X		X			
	17-item positive and negative affect scale	X	X	X		X	X
**Processes**	*Rehabilitation intensity*						
	Patient active time & actigraphy (at 20% of sessions at random)	collected each session throughout stay					
	Treatment engagement						
	Observer-rated Rehabilitation Participation Scale (at 20% of sessions at random)	collected each session throughout stay					
	Fidelity data (at 20% of session at random)	collected each session throughout stay					
	Post-Treatment Affect						
	Self-Assessment Moniker (at 20% of session at random)	collected each session throughout stay					
	Patient Satisfaction and Treatment Fidelity Survey	Collected after therapy sessions throughout stay					
	Other rehabilitation variables						
	Rehospitalization			X	X	X	X

	Disposition (home, long-term care)	X		X	X	X	X
	Social Participation (PROMIS Ability to Participate in Social Roles and Activities)					X	X
	Instrumental Activities of Daily Living (OARS IADL)					X	X
	Executive Functioning (Clock Drawing Test)	X					
	Fear of Falling	X					
	Readiness for Rehab	X	X				
	Short Blessed Test	X					
	Medical Comorbidity (CIRS-G)	X					
	Barthel Index: Pre-morbid version	X					

F3 SNF Admit (Baseline) Visit

The baseline visit will take place immediately or soon after the patient consents to the study. The rater will conduct the following assessments: Barthel, 6 minute walk, gait speed, SCID, MADRS, PANAS, Disposition, Clock Drawing Test, Fear of Falling, Readiness for Rehab, CIRS-G, Barthel Index: Pre-morbid version.

F4 Day 7 Visit

The day 7 visit will take place 7 days after the patient consents to the study. The rater will conduct the following assessments: PANAS, Readiness for Rehab.

F5 Day 30 Visit

The day 30 visit will take place 30 days after the patient's date of admission to the facility. If the patient has already been discharged from the skilled nursing facility, the rater will call the patient to schedule a home visit to conduct the following assessments: Barthel, gait speed, MADRS, PANAS, Follow-up Disposition.

F6 SNF Discharge

The discharge visit will take place as close in time to the patient's discharge date as possible. The rater will conduct the following assessments at the skilled nursing facility: Barthel, 6 minute walk, gait speed, Discharge Location..

F7 Day 60 Visit

The day 60 visit will take place 60 days after the patient's date of admission to the facility Many participants will be discharged from the skilled nursing facility, but others will still be residing there. Unblinded research staff will determine where this assessment is to be conducted on a case-by-case basis depending on the patient's status. These assessments may be conducted over the phone depending on the patient's ability. The rater will conduct the following assessments: Barthel, PANAS, Follow-up Disposition, PROMIS Ability to Participate in Social Roles and Activities, OARS IADL.

F8 Day 90 Visit

The day 90 visit will take place 90 days after the patient's date of admission to the facility. Many participants will be discharged from the skilled nursing facility, but others will still be residing there. Unblinded research staff will determine where this assessment is to be conducted on a case-by-case basis depending on the patient's status. The rater will conduct the following assessments: Barthel, PANAS, Follow-up Disposition, PROMIS Ability to Participate in Social Roles and Activities, OARS IADL.

F9 Data and Safety Monitoring Plan

The Data Safety Monitoring Plan (DSMP) outlined below has been approved by the NIMH.

9.a General description of monitoring plan: This is a single-site study. Dr. Lenze will be the primary monitoring entity. He will have primary responsibility for the monitoring of subjects during the entire time they participate in the study, both with respect to their safety (including confidentiality) and the integrity of their research data.

9.b Protection of Subject Privacy

In addition to research measures, clinical information obtained at initial evaluation and clinical treatment notes will become part of subjects' medical records. The PI and his staff will provide adequate safeguards for the protection of confidentiality of these records. Procedures designed to maintain confidentially include: (1) training for all research staff emphasizing the importance of confidentiality; (2) specific procedures developed to protect subjects' confidentiality, and (3) formal mechanisms limiting access to information that can link data to individual subjects. Data forms that include identifying information will be kept in locked cabinets. Only the unique ID number, assigned by the research coordinator at the time of initial contact will represent subjects during data entry, data transfer, data analysis, or other file management procedures. To facilitate tracking, a password-protected computer file will be maintained containing the identity of subjects, their ID numbers, and information about how they can be reached. This file, however, will contain no clinical data. Only members of the investigative group will have access to secured files or to master lists for subject code numbers and will be well informed regarding the protection of patients' rights to confidentiality. Identities of participants will not be revealed in the publication or presentation of any results from this project.

Videotaping permission and confidentiality: subjects will be videotaped for the purposes of observing the therapists and providing training material. Subjects will sign a consent which indicates the use of the videotaping, who may have access to the videotaping, and the length of time that the videotape will be kept by investigators. Videotapes will be kept with the same confidentiality procedures as other research data.

9.c Database Protection

All subject research data is stored separate from subject contact information, which is stored on our secure servers with network and database level passwords, only accessible to research staff who need contact with the subjects.

9.d Confidentiality During Adverse Event (AE) Reporting

AE reports and annual summaries will not include subject- or group-identifiable material. Each report will only include the identification code.

9.e Definitions of Adverse Events

An adverse event (AE) is any untoward medical occurrence in a subject temporally associated with participation in the clinical study or with use of the experimental agent being studied. An adverse finding can include a sign, symptom, abnormal assessment (laboratory test value, vital signs, electrocardiogram finding, etc.), or any combination of these.

A Serious Adverse Event (SAE) is any adverse event that results in one or more of the following outcomes:

- Death
- A life-threatening event
- Inpatient hospitalization or prolongation of existing hospitalization
- A persistent or significant disability/incapacity
- A congenital anomaly or birth defect
- Important medical event based upon appropriate medical judgment

9.f Classification of AE Severity

AEs will be labeled according to severity, which is based on their impact on the patient. An AE will be termed "mild" if it does not have a major impact on the patient, "moderate" if it causes the patient some minor inconvenience, and "severe" if it causes a substantial disruption to the patient's well being.

i AE Attribution Scale

AEs will be categorized according to the likelihood that they are related to the study intervention or other study procedures. Specifically, they will be labeled definitely unrelated, definitely related, probably related, or possibly related to the study intervention or procedures.

ii Severity

AEs will be labeled according to severity, which is based on their impact on the patient. An AE will be termed "mild" if it does not have a major impact on the patient, "moderate" if it causes the patient some minor inconvenience, and "severe" if it causes a substantial disruption to the patient's well being.

iii Expected Risks

Study assessments: The assessments to be conducted as part of this study are non-invasive and carry with them no more than minimal risk. The most significant risks to the subjects related to assessments are those that would follow a breach of confidentiality and the disclosure of clinical information or video recordings. In

addition, we recognize that medically ill and debilitated older adults can easily feel overwhelmed or fatigued by what would be minimally demanding tasks for others. Therefore, we include this discomfort in the potential risks. The instruments to be used in this study have been used by the PI in other studies of older medically ill and disabled adults. There are very few older adults who are unable to tolerate the length of the questionnaires. If this occurs, an assessment battery can be divided into several sessions.

Enhanced Medical Rehabilitation: Participants assigned to Enhanced Medical Rehabilitation will receive a higher intensity of therapy. This has the theoretical risk of causing cardiac events (as does any rehabilitation). Only trained physical and occupational therapists (and assistants), who are well-trained in the assessment of cardiac concerns during rehabilitation, will work with the participants.

Because this study involves participants with depression, there is a risk of suicidal behavior. However, these are risks associated with the illness not the study.

Risks in relation to benefits: No subjects will be denied interventions; participants receive either the standard of care rehabilitation, or Enhanced Medical Rehabilitation. No risks other than possible emotional distress exist from the study assessments. Thus, the risks involved in participating in this study are deemed low, which justifies the data and safety monitoring plan described here.

9.g Data Collection Procedures for Adverse Events

We will systematically collect AEs and summarize them in a running table throughout the study, including date of onset/offset, type of AE, severity, and interventions if any. Per Washington University IRB policy, reportable AEs are those that are possibly, probably, or definitely related to the study intervention or procedures.

9.h Reporting Procedures

SAEs that are unanticipated, serious, and possibly related to the study intervention will be reported to the IRB, and NIMH in accordance with requirements.

- Unexpected fatal or life-threatening AEs related to the intervention will be reported to the NIMH Program Officer within 7 days. Other serious and unexpected AEs related to the intervention will be reported to the NIMH Program Official within 15 days.
- Anticipated SAEs will be handled in a less urgent manner but will be reported to the IRB in accordance with their requirements.

G Statistical Plan

G1 Sample Size Determination and Power

Our sample size is based on 80% power to detect a Cohen's d=0.4 effect size on the ANCOVA difference in treatments at day 21, based on a two-tailed p<0.025 (because two outcome measures are used). G*Power 3.1.2 was used for power calculations. A Cohen's d=0.4 is between "small" (0.2) and "medium" (0.5) effect sizes. Effect sizes for function were in the "large" range, d=0.7-0.9, but we acknowledge that these observed

effect sizes are qualified by the small sample size, and it makes most sense to be more cautious regarding likely effect sizes in a full-scale study, hence our choice to power at d=0.4. Such an effect size is clinically relevant for the functional outcomes.[3] *We will only examine affective recovery as an outcome for those who are "affectively impaired" to begin with; i.e., those with clinical depression at baseline; with this reduced N, we are powered at 80% for a d=0.54. This same power analysis would apply for any subgroup analysis of depressed; i.e., if we wish to demonstrate that EMR is effective both in the case of clinical depression and in "non-depressed" older adults.*

G2 Interim Monitoring and Early Stopping

Not applicable; we do not have any interim data analysis or early stopping plans.

G3 Analysis Plan

The continuous demographic and clinical variables at baseline will be compared between the EMR group and the SOC group using the Two Independent Sample t Test or the Wilcoxon Two Sample Test, whichever is appropriate. Baseline categorical variables (e.g., race) will be compared between the groups using either the Chi Square Test or Fisher's Exact Test, whichever is appropriate. We will examine data descriptively using crosstabulations, histograms, and tests for normality (with corrective actions, data transformation or nonparametric alternatives, as needed). An analysis-appropriate technique will handle missing data.

G4 Statistical Methods

Time course analyses will be conducted to investigate the differences between pre and post outcome measures. These analyses will be used to ascertain whether there is a statistically significant difference in change over time between the EMR group and the SOC group, controlling for baseline values. Analyses of the outcome measures for normality will be carried out. Time course analysis techniques such as mixed effects models, t test of the differences, Wilcoxon test of the differences, or ANCOVA on the differences (using baseline value as the covariate) will then be conducted, depending on the normality tests. Our favored method for the primary analysis of baseline to day 30 (i.e., pre-post treatment) effects is the ANCOVA approach, comparing the EMR and standard-of-care groups in change scores, covarying for baseline scores. This approach is recommended as the superior and most well-powered approach for examining pre-post RCT data.[91] Our tests of the pilot data agreed with this: the ANCOVA approach had as much or more statistical power than other approaches (t-test, Wilcoxon, mixed-effect model) for all outcomes.

Participants will be randomized within each facility (i.e., stratified by facility) and then receive a PT/OT therapist pair who either is or is not trained/supervised in EMR based on randomization assignment. Thus participants are nested within therapists and therapists are nested within facilities. This multi-stage nested (hierarchical) design structure introduces possible correlation of participant outcomes with therapist. Such classification effects (facility/therapist) will be analyzed using Proc Nested in SAS or with the multi-level modeling available in Mplus that deals with stratification, clustering, and weighting.

We will also examine all time points to Day 90, to provide a real-world estimate of EMR and improve power to detect important secondary outcomes such as rehospitalization and institutionalization. We acknowledge these data may become somewhat naturalistic

in a rehabilitation population, given differences in length of stay, transfers to different facilities, and re-hospitalizations. Mixed effects models are the preferred method for these multiple-time-point analyses, especially as some missing data are to be expected post-discharge.[92]

H Data Handling and Record Keeping

H1 Confidentiality and Security

Procedures designed to maintain confidentially include: (1) formal training sessions for all research staff emphasizing the importance of confidentiality; (2) specific procedures developed to protect subjects' confidentiality, and (3) formal mechanisms limiting access to information that can link data to individual subjects. Data forms that include identifying information will be kept in locked cabinets. Only the unique ID number, assigned by the research coordinator at the time of initial contact will represent subjects during data entry, data transfer, data analysis, or other file management procedures. To facilitate tracking, a password-protected computer file will be maintained containing the identity of subjects, their ID numbers, and information about how they can be reached. This file, however, will contain no clinical data. Only members of the investigative group will have access to secured files or to master lists for subject code numbers and will be well informed regarding the protection of patients' rights to confidentiality. Identities of participants will not be revealed in the publication or presentation of any results from this project.

Videotaping permission and confidentiality: subjects will be videotaped for the purposes of observing the therapists and providing training material. Subjects will sign a consent which indicates the use of the videotaping, who may have access to the videotaping, and the length of time that the videotape will be kept by investigators. Videotapes will be kept with the same confidentiality procedures as other research data.

H2 Training

All research staff will receive standardized training in privacy and confidentiality. All research staff will be thoroughly trained on study procedures and assessments. Raters will role play assessments with supervisors. New raters will be observed by their supervisors before conducting assessments independently.

Therapists at partnering facilities interested in delivering the study intervention will receive training in human subjects research in accordance with the Washington University policy. They will also participate in initial and ongoing training in the study intervention.

H3 Records Retention

Records will be kept from all consented participants. De-identified information will be kept on excluded participants, limited to age, gender, race, and reason for exclusion.

I Study Monitoring, Auditing, and Inspecting

I1 Study Monitoring Plan

As per data and safety monitoring plan above, which has been approved by the NIMH. There is no additional monitoring for this study.

I2 Auditing and Inspecting

Not applicable.

J Study Administration

J1 Organization and Participating Centers

Research staff include Washington University faculty and staff. Recruitment and study intervention will take place at Barnes Jewish Extended Care and Alexian Brothers Sherbrooke Village.

Prior to recruiting patients for the study, the research team may work with partnering and potential partnering facilities to gather information about institutional practice in order to a) facilitate the development of operating procedures b) ensure that the intervention is not already being conducted there.

J2 Funding Source and Conflicts of Interest

This study is funded by The National Institute of Mental Health (NIMH). No members of the study team have any conflicts of interest.

J3 Participant Payment

Participants will be paid $100 in the form of a check. Payment will not be pro-rated for early withdrawal.

K Publication Plan

We plan to publish findings in a peer-reviewed journal in a prompt manner as soon as the study is completed.

L References

1. Hall MJ, DeFrances CJ, Williams SN, Golosinskiy A, Schwartzman A. National Hospital Discharge Survey: 2007 summary. *National health statistics reports.* Oct 26 2010(29):1-20, 24.
2. Hardy SE, Gill TM. Recovery from disability among community-dwelling older persons. *JAMA.* Apr 7 2004;291(13):1596-1602.
3. Alley DE, Hicks GE, Shardell M, et al. Meaningful improvement in gait speed in hip fracture recovery. *J Am Geriatr Soc.* Sep 2011;59(9):1650-1657.
4. Crimmins EM, Saito Y. Getting better and getting worse. *Journal of Aging and Health.* 1993;5(1):3-36.
5. Kaplan SJ. Growth and payment adequacy of medicare postacute care rehabilitation. *Arch Phys Med Rehab.* Nov 2007;88(11):1494-1499.
6. Fredman L, Hawkes WG, Black S, Bertrand RM, Magaziner J. Elderly patients with hip fracture with positive affect have better functional recovery over 2 years. *J Am Geriatr Soc.* Jul 2006;54(7):1074-1081.
7. Lequerica AH, Donnell CS, Tate DG. Patient engagement in rehabilitation therapy: physical and occupational therapist impressions. *Disability and rehabilitation.* 2009;31(9):753-760.
8. Alexopoulos GS, Sirey JA, Raue PJ, Kanellopoulos D, Clark TE, Novitch RS. Outcomes of depressed patients undergoing inpatient pulmonary rehabilitation. *Am J Geriatr Psychiatry.* May 2006;14(5):466-475.
9. Burns A, Banerjee S, Morris J, et al. Treatment and prevention of depression after surgery for hip fracture in older people: randomized, controlled trials. *J Am Geriatr Soc.* Jan 2007;55(1):75-80.
10. Barth J, Paul J, Harter M, Bengel J. Inpatient psychotherapeutic treatment for cardiac patients with depression in Germany - short term results. *Psychosoc Med.* 2005;2:Doc04.
11. Pollock BG. Adverse reactions of antidepressants in elderly patients. *The Journal of clinical psychiatry.* 1999;60 Suppl 20:4-8.
12. Ormel J, Rijsdijk FV, Sullivan M, van Sonderen E, Kempen GI. Temporal and reciprocal relationship between IADL/ADL disability and depressive symptoms in late life. *J Gerontol B Psychol Sci Soc Sci.* Jul 2002;57(4):P338-347.
13. Lenze EJ, Rogers JC, Martire LM, et al. The association of late-life depression and anxiety with physical disability: a review of the literature and prospectus for future research. *Am J Geriatr Psychiatry.* Spring 2001;9(2):113-135.
14. Incalzi RA, Cesari M, Pedone C, Carbonin PU. Construct validity of the 15-item geriatric depression scale in older medical inpatients. *Journal of geriatric psychiatry and neurology.* Mar 2003;16(1):23-28.
15. Phifer JF, Murrell SA. Etiologic factors in the onset of depressive symptoms in older adults. *Journal of abnormal psychology.* Aug 1986;95(3):282-291.
16. Barry LC, Allore HG, Bruce ML, Gill TM. Longitudinal association between depressive symptoms and disability burden among older persons. *J Gerontol A Biol Sci Med Sci.* Dec 2009;64(12):1325-1332.
17. NIH Workgroup. Advancing The Science of Effective Behavioral Treatments in Primary Care. 2010; http://conferences.thehillgroup.com/OBSSR/IntegratedHealth/about.html.
18. Talkowski JB, Lenze EJ, Munin MC, Harrison C, Brach JS. Patient participation and physical activity during rehabilitation and future functional outcomes in patients after hip fracture. *Arch Phys Med Rehab.* Apr 2009;90(4):618-622.

19. Lang CE, Macdonald JR, Reisman DS, et al. Observation of amounts of movement practice provided during stroke rehabilitation. *Arch Phys Med Rehab.* Oct 2009;90(10):1692-1698.
20. Lequerica AH, Kortte K. Therapeutic engagement: a proposed model of engagement in medical rehabilitation. *American journal of physical medicine & rehabilitation / Association of Academic Physiatrists.* May 2010;89(5):415-422.
21. Prochaska JO, Velicer WF. The transtheoretical model of health behavior change. *Am J Health Promot.* Sep-Oct 1997;12(1):38-48.
22. Lenze EJ, Munin MC, Quear T, et al. Significance of poor patient participation in physical and occupational therapy for functional outcome and length of stay. *Arch Phys Med Rehabil.* Oct 2004;85(10):1599-1601.
23. Skidmore ER, Whyte EM, Holm MB, et al. Cognitive and affective predictors of rehabilitation participation after stroke. *Arch Phys Med Rehabil.* Feb 2010;91(2):203-207.
24. Lenze EJ, Munin MC, Dew MA, et al. Adverse effects of depression and cognitive impairment on rehabilitation participation and recovery from hip fracture. *Int J Geriatr Psychiatry.* May 2004;19(5):472-478.
25. Frederick JT, Steinman LE, Prohaska T, et al. Community-based treatment of late life depression an expert panel-informed literature review. *Am J Prev Med.* Sep 2007;33(3):222-249.
26. Hershkovitz A, Kalandariov Z, Hermush V, Weiss R, Brill S. Factors affecting short-term rehabilitation outcomes of disabled elderly patients with proximal hip fracture. *Arch Phys Med Rehabil.* Jul 2007;88(7):916-921.
27. Lenze EJ, Skidmore ER, Dew MA, et al. Does depression, apathy or cognitive impairment reduce the benefit of inpatient rehabilitation facilities for elderly hip fracture patients? *Gen Hosp Psychiatry.* Mar-Apr 2007;29(2):141-146.
28. Morghen S, Bellelli G, Manuele S, Guerini F, Frisoni GB, Trabucchi M. Moderate to severe depressive symptoms and rehabilitation outcome in older adults with hip fracture. *Int J Geriatr Psychiatry.* Nov 9 2010.
29. Oude Voshaar RC, Banerjee S, Horan M, et al. Fear of falling more important than pain and depression for functional recovery after surgery for hip fracture in older people. *Psychol Med.* Nov 2006;36(11):1635-1645.
30. Melvin JL. Standards For Assessing Medical Appropriateness Criteria For Admitting Patients To Rehabilitation Hospitals or Units (www.aapmr.org).
31. Gateway Rehabilitation Network. Inpatient Rehab Referral Guidelines (webpage: www.gtarehabnetwork.ca).
32. Buntin MB. Access to postacute rehabilitation. *Arch Phys Med Rehabil.* Nov 2007;88(11):1488-1493.
33. Bandura A. Self-efficacy: toward a unifying theory of behavioral change. *Psychol Rev.* Mar 1977;84(2):191-215.
34. Rasmussen HN, Wrosch C, Scheier MF, Carver CS. Self-regulation processes and health: the importance of optimism and goal adjustment. *J Pers.* Dec 2006;74(6):1721-1747.
35. Ajzen I. The theory of planned behavior. *Organizational behavior and human decision processes.* 1991;50(2):179-211.
36. Baum CM, Edwards DF. *The Washington University Activity Card Sort*. St. Louis: PenUltima Press; 2001.
37. Nelson ME, Rejeski WJ, Blair SN, et al. Physical activity and public health in older adults: recommendation from the American College of Sports Medicine and the American Heart Association. *Med Sci Sports Exerc.* Aug 2007;39(8):1435-1445.

38. Missiuna C, Mandich AD, Polatajko HJ, Malloy-Miller T. Cognitive orientation to daily occupational performance (CO-OP): part I--theoretical foundations. *Phys Occup Ther Pediatr.* 2001;20(2-3):69-81.
39. Ylvisaker M, Turkstra L, Coehlo C, et al. Behavioural interventions for children and adults with behaviour disorders after TBI: a systematic review of the evidence. *Brain Inj.* Jul 2007;21(8):769-805.
40. Hildebrand MW, Host HH, Binder EF, et al. Measuring treatment fidelity in a rehabilitation intervention study. *Am J Phys Med Rehabil.* 2012: 91: 715-724.
41. Whyte J, Hart T. It's more than a black box; it's a Russian doll: defining rehabilitation treatments. *American journal of physical medicine & rehabilitation / Association of Academic Physiatrists.* Aug 2003;82(8):639-652.
42. Allen NE, Sherrington C, Suriyarachchi GD, Paul SS, Song J, Canning CG. Exercise and motor training in people with Parkinson's disease: a systematic review of participant characteristics, intervention delivery, retention rates, adherence, and adverse events in clinical trials. *Parkinsons Dis.* 2012; 85(4): 3-28.
43. Bovend'Eerdt TJ, Dawes H, Sackley C, Izadi H, Wade DT. An integrated motor imagery program to improve functional task performance in neurorehabilitation: a single-blind randomized controlled trial. *Arch Phys Med Rehab.* Jun 2010;91(6):939-946.
44. Waltz J, Addis ME, Koerner K, Jacobson NS. Testing the integrity of a psychotherapy protocol: assessment of adherence and competence. *J Consult Clin Psychol.* Aug 1993;61(4):620-630.
45. Perepletchikova F, Treat TA, Kazdin AE. Treatment integrity in psychotherapy research: analysis of the studies and examination of the associated factors. *J Consult Clin Psychol.* Dec 2007;75(6):829-841.
46. Lenze EJ, Host HH, Hildebrand M, et al. Enhanced Medical Rehabilitation Is Feasible in a Skilled Nursing Facility: Preliminary Data on a Novel Treatment for Older Adults With Depression. *The American journal of geriatric psychiatry : official journal of the American Association for Geriatric Psychiatry.* Jul 20 2011.
47. Lenze EJ, Host HH, Hildebrand MW, et al. Enhanced medical rehabilitation increases therapy intensity and engagement and improves functional outcomes in postacute rehabilitation of older adults: a randomized-controlled trial. *J Am Med Dir Assoc.* Oct 2012;13(8):708-712.
48. Hildebrand MW, Host HH, Binder EF, et al. Measuring Treatment Fidelity in a Rehabilitation Intervention Study. *American journal of physical medicine & rehabilitation / Association of Academic Physiatrists.* Feb 28 2012.
49. Binder EF, Brown M, Sinacore DR, Steger-May K, Yarasheski KE, Schechtman KB. Effects of extended outpatient rehabilitation after hip fracture: a randomized controlled trial. *JAMA.* Aug 18 2004;292(7):837-846.
50. Holliday RC, Ballinger C, Playford ED. Goal setting in neurological rehabilitation: patients' perspectives. *Disabil Rehabil.* Mar 15 2007;29(5):389-394.
51. Kortte KB, Falk LD, Castillo RC, Johnson-Greene D, Wegener ST. The Hopkins Rehabilitation Engagement Rating Scale: development and psychometric properties. *Arch Phys Med Rehabil.* Jul 2007;88(7):877-884.
52. Wressle E, Eeg-Olofsson AM, Marcusson J, Henriksson C. Improved client participation in the rehabilitation process using a client-centred goal formulation structure. *J Rehabil Med.* Jan 2002;34(1):5-11.
53. Jette DU, Warren RL, Wirtalla C. Rehabilitation in skilled nursing facilities: effect of nursing staff level and therapy intensity on outcomes. *American journal of*

physical medicine & rehabilitation / Association of Academic Physiatrists. Sep 2004;83(9):704-712.
54. Lenze EJ, Munin MC, Quear T, et al. The Pittsburgh Rehabilitation Participation Scale: reliability and validity of a clinician-rated measure of participation in acute rehabilitation. *Arch Phys Med Rehabil.* Mar 2004;85(3):380-384.
55. Horvath AO, Greenberg LS. Development and Validation of the Working Alliance Inventory. *J Couns Psychol.* Apr 1989;36(2):223-233.
56. Hall AM, Ferreira PH, Maher CG, Latimer J, Ferreira ML. The influence of the therapist-patient relationship on treatment outcome in physical rehabilitation: a systematic review. *Physical therapy.* Aug 2010;90(8):1099-1110.
57. Raue PJ, Goldfried MR, Barkham M. The therapeutic alliance in psychodynamic-interpersonal and cognitive-behavioral therapy. *J Consult Clin Psychol.* Aug 1997;65(4):582-587.
58. Thorpe KE, Zwarenstein M, Oxman AD, et al. A pragmatic-explanatory continuum indicator summary (PRECIS): a tool to help trial designers. *Journal of clinical epidemiology.* May 2009;62(5):464-475.
59. Glasgow RE, Vogt TM, Boles SM. Evaluating the public health impact of health promotion interventions: the RE-AIM framework. *American journal of public health.* Sep 1999;89(9):1322-1327.
60. Koepsell TD, Zatzick DF, Rivara FP. Estimating the population impact of preventive interventions from randomized trials. *Am J Prev Med.* Feb 2011;40(2):191-198.
61. Marcus SM. A sensitivity analysis for subverting randomization in controlled trials. *Stat Med.* Feb 28 2001;20(4):545-555.
62. Coleman SA, Cunningham CJ, Walsh JB, et al. Outcomes among older people in a post-acute inpatient rehabilitation unit. *Disability and rehabilitation.* Dec 8 2011.
63. Granger CV, Dewis LS, Peters NC, Sherwood CC, Barrett JE. Stroke rehabilitation: analysis of repeated Barthel index measures. *Arch Phys Med Rehab.* Jan 1979;60(1):14-17.
64. van der Putten JJ, Hobart JC, Freeman JA, Thompson AJ. Measuring change in disability after inpatient rehabilitation: comparison of the responsiveness of the Barthel index and the Functional Independence Measure. *Journal of neurology, neurosurgery, and psychiatry.* Apr 1999;66(4):480-484.
65. Collin C, Wade DT, Davies S, Horne V. The Barthel ADL Index: a reliability study. *Int Disabil Stud.* 1988;10(2):61-63.
66. Wade DT, Collin C. The Barthel ADL Index: a standard measure of physical disability? *Int Disabil Stud.* 1988;10(2):64-67.
67. al-Khawaja I, Wade DT, Turner F. The Barthel Index and its relationship to nursing dependency in rehabilitation. *Clin Rehabil.* Nov 1997;11(4):335-337.
68. Bond J, Gregson B, Smith M, Lecouturier J, Rousseau N, Rodgers H. Predicting place of discharge from hospital for patients with a stroke or hip fracture on admission. *J Health Serv Res Policy.* Jul 2000;5(3):133-139.
69. Hardy SE, Perera S, Roumani YF, Chandler JM, Studenski SA. Improvement in usual gait speed predicts better survival in older adults. *J Am Geriatr Soc.* Nov 2007;55(11):1727-1734.
70. Perera S, Mody SH, Woodman RC, Studenski SA. Meaningful change and responsiveness in common physical performance measures in older adults. *J Am Geriatr Soc.* May 2006;54(5):743-749.
71. Studenski S, Perera S, Patel K, et al. Gait speed and survival in older adults. *JAMA.* Jan 5 2011;305(1):50-58.

72. Alexopoulos GS, Abrams RC, Young RC, Shamoian CA. Use of the Cornell scale in nondemented patients. *Journal of the American Geriatrics Society.* Mar 1988;36(3):230-236.
73. Alexopoulos GS, Abrams RC, Young RC, Shamoian CA. Cornell Scale for Depression in Dementia. *Biological Psychiatry.* Feb 1 1988;23(3):271-284.
74. Debruyne H, Van Buggenhout M, Le Bastard N, et al. Is the geriatric depression scale a reliable screening tool for depressive symptoms in elderly patients with cognitive impairment? *Int J Geriatr Psychiatry.* Jun 2009;24(6):556-562.
75. Rosenberg PB, Drye LT, Martin BK, et al. Sertraline for the treatment of depression in Alzheimer disease. *Am J Geriatr Psychiatry.* Feb 2010;18(2):136-145.
76. Lenze EJ, Munin MC, Skidmore ER, et al. Onset of depression in elderly persons after hip fracture: implications for prevention and early intervention of late-life depression. *J Am Geriatr Soc.* Jan 2007;55(1):81-86.
77. Watson D, Clark LA, Tellegen A. Development and validation of brief measures of positive and negative affect: the PANAS scales. *Journal of Personality and Social Psychology.* Jun 1988;54(6):1063-1070.
78. Kercher K. Assessing subjective well-being in the old-old. *Research on Aging.* 1992;14(2):131-168.
79. Marteau TM, Bekker H. The development of a six-item short-form of the state scale of the Spielberger State-Trait Anxiety Inventory (STAI). *Br J Clin Psychol.* Sep 1992;31 (Pt 3):301-306.
80. Ostir GV, Smith PM, Smith D, Ottenbacher KJ. Reliability of the positive and negative affect schedule (PANAS) in medical rehabilitation. *Clin Rehabil.* Oct 2005;19(7):767-769.
81. De Wit L, Putman K, Baert I, et al. Anxiety and depression in the first six months after stroke. A longitudinal multicentre study. *Disabil Rehabil.* 2008;30(24):1858-1866.
82. Benzer W, Platter M, Oldridge NB, et al. Short-term patient-reported outcomes after different exercise-based cardiac rehabilitation programmes. *Eur J Cardiovasc Prev Rehabil.* Jun 2007;14(3):441-447.
83. Brenes GA. Anxiety and chronic obstructive pulmonary disease: prevalence, impact, and treatment. *Psychosom Med.* Nov-Dec 2003;65(6):963-970.
84. Dorra HH, Lenze EJ, Kim Y, et al. Clinically relevant behaviors in elderly hip fracture inpatients. *Int J Psychiatry Med.* 2002;32(3):249-259.
85. Sanislow CA, Pine DS, Quinn KJ, et al. Developing constructs for psychopathology research: research domain criteria. *J Abnorm Psychol.* Nov 2010;119(4):631-639.
86. First M, Spitzer R, Gibbon M, Williams J. *Structured Clinical Interview for DSM-IV Axis I Disorders (SCID)*. Washington, D.C.: American Psychiatric Press; 1997.
87. Kocsis JH, Gerber AJ, Milrod B, et al. A new scale for assessing the quality of randomized clinical trials of psychotherapy. *Comprehensive psychiatry.* May-Jun 2010;51(3):319-324.
88. Hildebrand M, Host HH, Kraus L, Lenze E, Binder EF. Novel methods for increasing and measuring occupational therapy intensity in the skilled nursing rehabilitation setting. Indianapolis, IN: Poster presentation at the 2012 AOTA National Conference in Indianapolis, IN; 2012.
89. Miller MD, Paradis CF, Houck PR, et al. Rating chronic medical illness burden in geropsychiatric practice and research: application of the Cumulative Illness Rating Scale. *Psychiatry Res.* Mar 1992;41(3):237-248.

90. Katzman R, Brown T, Fuld P, Peck A, Schechter R, Schimmel H. Validation of a short Orientation-Memory-Concentration Test of cognitive impairment. *The American journal of psychiatry.* Jun 1983;140(6):734-739.
91. Vickers AJ. Parametric versus non-parametric statistics in the analysis of randomized trials with non-normally distributed data. *BMC medical research methodology.* 2005;5:35.
92. Petkova E, Teresi J. Some statistical issues in the analyses of data from longitudinal studies of elderly chronic care populations. *Psychosom Med.* May-Jun 2002;64(3):531-547.
93. Baron RM, Kenny DA. The moderator-mediator variable distinction in social psychological research: conceptual, strategic, and statistical considerations. *Journal of Personality and Social Psychology.* Dec 1986;51(6):1173-1182.
94. Kraemer HC, Wilson GT, Fairburn CG, Agras WS. Mediators and moderators of treatment effects in randomized clinical trials. *Arch Gen Psychiatry.* Oct 2002;59(10):877-883.
95. Leon AC. Two clinical trial designs to examine personalized treatments for psychiatric disorders. *The Journal of clinical psychiatry.* May 2011;72(5):593-597.
96. Chan L. The state-of-the-science: Challenges in designing postacute care payment policy. *Arch Phys Med Rehab.* Nov 2007;88(11):1522-1525.
97. Muthén B, Asparouhov T. *Growth mixture modeling: Analysis with non-Gaussian random effects*. Boca Raton: Chapman, all/CRC Press; 2008.
98. Muthén B, Muthén L. Mplus User's Guide. In: Muthen M, ed. Los Angeles, CA2008.
99. Kohl PL, Kagotho N, Dixon D. Parenting Behaviors Among Depressed Mothers in the Child Welfare System. *Social Work Research.* 2011;35:215-225.
100. Chambless DL, Hollon SD. Defining empirically supported therapies. *Journal of Consulting and Clinical Psychology.* Feb 1998;66(1):7-18.
101. Fortney JC, Pyne JM, Smith JL, et al. Steps for implementing collaborative care programs for depression. *Popul Health Manag.* Apr 2009;12(2):69-79.
102. Herschell AD, Kolko DJ, Baumann BL, Davis AC. The role of therapist training in the implementation of psychosocial treatments: a review and critique with recommendations. *Clin Psychol Rev.* Jun 2010;30(4):448-466.
103. Salkever DS, Johnston S, Karakus MC, Ialongo NS, Slade EP, Stuart EA. Enhancing the net benefits of disseminating efficacious prevention programs: a note on target efficiency with illustrative examples. *Adm Policy Ment Health.* Jul 2008;35(4):261-269.
104. McHugh RK, Barlow DH. The dissemination and implementation of evidence-based psychological treatments. A review of current efforts. *The American psychologist.* Feb-Mar 2010;65(2):73-84.
105. Arean PA, Raue PJ, Sirey JA, Snowden M. Implementing evidence-based psychotherapies in settings serving older adults: challenges and solutions. *Psychiatric Services.* Jun 2012;63(6):605-607.
106. Kazdin AE. Evidence-based treatment research: Advances, limitations, and next steps. *The American psychologist.* Nov 2011;66(8):685-698.
107. Insel TR. Translating scientific opportunity into public health impact: a strategic plan for research on mental illness. *Arch Gen Psychiatry.* Feb 2009;66(2):128-133.

Enhanced Medical Rehabilitation in Older Adults (EMR R01)
Statistical Analysis Plan (Final)

Trial Registration Number: NCT02114879
SAP Version: 2
Protocol Version: 8

Roles and Responsibilities

Michael Yingling MS; SAP Author
Philip Miller AB; Senior Biostatistician
Eric Lenze, MD; Principal Investigator
Thomas Rodebaugh PhD; Co-Investigator
Emily Lenard, MSW; Study Coordinator

Abbreviations:

EMR: Enhanced Medical Rehabilitation

SOC: Standard of Care

SNF: Skilled Nursing Facility

OT: Occupational Therapy

PT: Physical Therapy

PI: Principal Investigator

AE: Adverse Event

SAE: Serious Adverse Event

MADRS: Montgomery and Asberg Depression Rating Scale.

CIRS-G: Cumulative Illness Rating Scale for Geriatrics

SBT: Short Blessed Test

SAS: Statistical Analysis Software

ANCOVA: Analysis of Covariance

Introduction:

i. Background and Rationale:

Americans are aging and having more medical events such as heart attack, stroke, and hip fracture. More than ever, older adults are surviving these events and leaving the hospital, alive but severely disabled. The solution for these highly disabled older adults is rehabilitation in the post-acute care setting, a large and rapidly-growing sector of care. Post-acute rehabilitation consists of daily physical therapy (PT) and occupational therapy (OT) to achieve restoration (e.g., regain walking ability and counteract bone and muscle loss by gait and muscle strengthening exercises), and adaptation (e.g., learn to perform activities of daily living and safely live at home). The most common setting for post-acute rehabilitation is a skilled nursing facility (SNF). Length of stay is about 3 weeks, a narrow window in which to recover and return home or be institutionalized.

Post-acute rehabilitation is low-intensity, low-engagement and too often fails to achieve functional recovery, especially in the most vulnerable individuals. This problem should be remediable by applying the science of behavior change to this setting. We therefore developed Enhanced Medical Rehabilitation.

ii. Objectives:

1. Aim 1 (Primary): Examine the effectiveness of EMR for improving functional outcomes in older adults admitted to SNFs for post-acute rehabilitation.

   H1: EMR will improve functional recovery to a greater extent than standard-of-care rehabilitation.

   Explanation: The purpose of H1 is to examine the effectiveness of EMR. If H1 is supported, this could establish EMR as the gold-standard practice for post-acute rehabilitation. Our primary analysis is the change from baseline to Discharge. This single primary endpoint allows for an effectiveness test that is not confounded by length of stay differences or post-discharge issues beyond the reach of EMR.

2. Aim 2: Examine EMR's ability to overcome patient-level barriers to successful rehabilitation.

   H2: The effectiveness of EMR for functional recovery will be greatest in: (a) patients with clinical depression; (b) patients with high levels of medical comorbidity; (c) patients with cognitive impairment.

   Explanation: EMR was designed to overcome barriers to rehabilitation (for example, due to depression); therefore, the difference in functional recovery between EMR and SOC should be greater in the most vulnerable older adults. If H2 is supported, we will demonstrate that older adults with these common comorbidities benefit the most from high intensity, high engagement rehabilitation. If so, this would refute conventional wisdom in post-acute rehabilitation, codified in admission criteria for rehabilitation facilities, regarding the appropriate candidates for rehabilitation (as described in Significance).

3. Aim 3 (Secondary): Effect of EMR on MADRS scores on individuals with clinical depression.

4. Calculated a rate of clinical depression (SCID for those with elevated MADRS score):

22 participants had the SCID administered.
8 did not have depression diagnosis while 14 did have depression diagnosis.

Conclusion: only 14 individuals in the study had a clinical depression (ie, a diagnosis of depression). No further analyses to be done.

<u>Additional Variables/Outcomes</u>

6-Minute Walk/Gait Speed: Administered at Baseline and Discharge. Two components for 6-Minute Walk:
1) Distance Ambulated (feet): text box. If unable to complete, distance was entered as a 0.
2) Time: options are 6 minutes, Other time (MM:SS), and N/A

Two components for Gait Speed (Baseline, Day 30, Discharge):
1) Distance: radio button (10 meters, 4 meters, 0 meters). If unable, 0 meters selected.
2) Time (seconds): text box (if unable, time is 0).

Disposition (i.e., discharge to community vs. not)

Rehospitalization (rate).

Secondary analyses (not for main paper):
1. PANAS (A positive and a negative affect score).

2. PROMIS (Ability to Participant in Social Roles and Activities).
3. OARS IADL.

Process data: Measures of the dosage of intervention (# of EMR steps done in each therapy activity; PAT %; Rehab Participation Scale Score) before and after training.

<u>Study Methods:</u>

i. Trial Design:

This was a randomized, parallel-group trial where patients received either EMR or SOC therapy.

Patients who received EMR received therapy only from therapists who were trained and supervised in EMR. Their therapy sessions followed the EMR protocol. Otherwise, their SNF care did not differ from usual care.

Patients who received SOC received therapy only from PT/OTs (or assistants) who were not trained nor supervised in EMR. These therapists were monitored (videotaped or observed) but were not asked to do anything differently with their patients.

ii. Randomization:

Randomization (blocks…2 and 4 patients). was 1:1 to EMR or SOC with stratification by SNF site and by baseline depression status.

iii. Sample Size:

The sample size was based on 80% power to detect a Cohen's d=0.4 effect size on the ANCOVA difference in treatments at primary endpoint, based on a two-tailed p<0.025 (because two outcome measures are used). G*Power 3.1.2 was used for power calculations. A Cohen's d=0.4 is between "small" (0.2) and "medium" (0.5) effect sizes. Effect sizes for function were in the "large" range, d=0.7-0.9, but we acknowledge that these observed effect sizes are qualified by the small sample size, and it makes most sense to be more cautious regarding likely effect sizes in a full-scale study, hence our choice to power at d=0.4. Such an effect size is clinically relevant for the functional outcomes. *We will only examine affective recovery as an outcome for those who are "affectively impaired" to begin with; i.e., those with clinical depression at baseline; with this reduced N, we are powered at 80% for a d=0.54. This same power analysis would apply for any subgroup analysis of depressed; i.e., if we wish to demonstrate that EMR is effective both in the case of clinical depression and in "non-depressed" older adults.*

iv. Statistical Interim Analyses/Stopping Guidance:

Not applicable; we did not have any interim data analysis or early stopping plans.

v. Timing of Final Analysis:

All outcomes will be analyzed collectively.

vi. Timing of Outcome Assessments (including windows):

	**Schedule of Assessments**	**SNF admit (baseline)**	**Day 7**	**Day 30**	**SNF discharge**	**Day 60**	**Day 90**
**tco me**	<u>*Function*</u>						

	**Barthel Index (primary outcome)**	X		X	X	X	X
	Gait speed	X		X	X		
	6-minute walk	X			X		
	Depression SCID (given if indicated by MADRS)	X					
	**MADRS**	X		X			
	17-item positive and negative affect scale	X	X	X		X	X
**Processes**	*Rehabilitation intensity*						
	Patient active time & actigraphy (at 20% of sessions at random)	collected each session throughout stay					
	Treatment engagement						
	Observer-rated Rehabilitation Participation Scale (at 20% of sessions at random)	collected each session throughout stay					
	Fidelity data (at 20% of session at random)	collected each session throughout stay					
	Post-Treatment Affect						
	Self-Assessment Moniker (at 20% of session at random)	collected each session throughout stay					
	Patient Satisfaction and Treatment Fidelity Survey	Collected after therapy sessions					
	Other rehabilitation variables						
	Rehospitalization			X	X	X	X
	Disposition (home, long-term care)	X		X	X	X	X
	Social Participation (PROMIS Ability to Participate in Social Roles and Activities)					X	X
	Instrumental Activities of Daily Living (OARS IADL)					X	X
	Executive Functioning (Clock Drawing Test)	X					
	Fear of Falling	X					
	Readiness for Rehab	X	X				
	Short Blessed Test	X					
	Medical Comorbidity (CIRS-G)	X					
	Barthel Index: Pre-morbid version	X					

Statistical Principles:

i. Confidence Intervals and P-Values

Level of significance is 5%

ii. Adherence and protocol deviations

Protocol Deviation:

Any alteration or modification to the IRB-approved research without prospective IRB approval. The term research encompasses all IRB-approved materials and documents including the detailed protocol, IRB application, consent form, recruitment materials, questionnaires/data collection forms, and any other information relating to the research study.

Therapist adherence and competence was tracked two times per participant.

Variables: number of EMR principles used per therapy session; PAT.

iii. Analysis Populations

Trial Population:

i. Screening data to describe representativeness of trial sample will include age, gender, race and ethnicity.

ii. Eligibility

Inclusion Criteria:

a. 65 years of age and older
b. Admitted to a skilled nursing facility for post-acute care from PT and OT for 2 weeks or more.

Exclusion Criteria:

a. Language, visual or hearing barriers to participation (e.g. unable to communicate with research staff).
b. Medical illness preventing study participation or accurate data collection (e.g., highly unstable cardiac illness such that early re-hospitalization is expected; metastatic or other cancer such that hospice is recommended or survival is limited.
c. Moderate-severe dementia (demonstrated by chart diagnosis and/or short blessed score greater than 13).
d. Progressive neurological condition such that recovery of function is not feasible.
e. Patient did not have the ability to walk prior to hospitalization (e.g. paraplegic).
f. Schizophrenia or other chronic or current psychotic disorder.
g. Patient normally resides in a long-term care facility.

iii. Recruitment: the numbers that will be presented in a CONSORT diagram include:

1. # Screened/Consented and reasons for ineligibility.

2. # Randomized to EMR vs SOC.
3. # Present at each follow-up time point (i.e., Day 30, Discharge, Day 60, and Day 90); reasons for discontinuation/lost to follow-up.
4. # Included in Final Analysis; reasons excluded from final analysis.

iv. Withdrawal/follow-up

No follow-up was done for withdrawn participants. If a participant was withdrawn, then an attempt was made to obtain final prognosis information using clinical data available from the chart unless explicitly told not to do so by the participant.

v. Baseline Patient Characteristics

In addition to the screening variables described above, the variables below will also be summarized in a Baseline Characteristics Table. Continuous variables will be summarized using median and IQR while categorical variables will be summarized using counts and percentages. These statistics will be provided by condition (EMR Vs SOC) and as a whole.

- Barthel Index
- MADRS Score
- Gait Speed (Distance in meters and Time in seconds)
- 6-Minute Walk (Distance Ambulated, in feet)
- PANAS Score
- Short Blessed Test Score
- CIRS-G Score
- Clock Drawing Score

<u>Analysis:</u>

i. Outcome Definitions

1) Aim 1: Examine the effectiveness of EMR for improving functional outcomes in older adults admitted to SNFs for post-acute rehabilitation.

H1: EMR will improve functional recovery to a greater extent than standard-of-care rehabilitation.

The primary measure employed for assessing functional improvement will be The Barthel Index. This index is a 10-item scale used to ascertain the degree of independence of normal daily activities. This will be done by taking the sum of

these 10 items. A high score indicates a high degree of independence, and a score can range from 0 to 100.

2) Aim 2: Examine EMR's ability to overcome patient-level barriers to successful rehabilitation.

H2: The effectiveness of EMR for functional recovery will be greatest in: (a) patients with clinical depression; (b) patients with high levels of medical comorbidity; (c) patients with cognitive impairment.

Part (a) will be assessed using the MADRS, which is a 10-item scale used to assess for major depression. Each item is rated on a scale of 0 to 6, where a 6 is worse. The total score (i.e., sum of these 10 items) will be used to assess for major depression and can range from 0 to 60.

Part (b) will be assessed using the CIRS-G, which is a 13-item scale used to assess for disease in all major body systems. Each item is rated on a scale of 0 to 4, with a 4 indicating severe impairment. The total score (i.e., sum of these 10 items) will be used to assess for medical comorbidity and can range from 0 to 52.

Part (c) will be assessed using the SBT Scores. The SBT is a 6-item scale used to assess for memory/concentration deficits. A total score, which is obtained after applying a weighting factor to each item then summing up the final item scores, can range from 0 to 28. A high score indicates impairment consistent with dementia.

ii. Analysis Methods

The primary outcome analysis for functional recovery will use a marginal model with time (baseline and discharge), condition (E-MR vs. SOC) and time x condition as fixed effects and with a covariance structure specified based on the Bayesian Information Criterion (BIC). Employing a marginal model allows for a more robust approach in the handling of missing data compared to the traditional ANCOVA model.

For H2 under Aim 2, each potential continuous moderator will be individually inserted into the primary model. The term of interest will be the time x condition x moderator interaction.

The secondary analyses for Six-Minute Walk distance and Gait Speed will use a Mann-Whitney U Test to test for differences in means between groups. In

addition, a Chi-Square test will be employed to test for independence between condition and the secondary outcome variable re-hospitalization. Finally, the outcome of self-reported function will use a marginal model using time (30, 60, and 90 days), condition and time x condition as fixed effects, with a covariance structure specified based on BIC.

Exploratory analyses will also test whether the effects of age, gender, race and site (i.e., Barnes Jewish Extended Care Vs Alexian Brothers Sherbrooke Village) altered the conclusions of the primary results as well as the conclusions of the moderator results.

Note: we will not look at the nesting of subjects within therapists who are then nested within site as this is not possible due to the inconsistency of assigning therapists to participants.

iii. Missing Data

We use a marginal model which accounts for missing data (no multiple imputation was used).

iv. Harms

An adverse event (AE) is any untoward medical occurrence in a subject temporally associated with participation in the clinical study or with use of the experimental agent being studied. An adverse finding can include a sign, symptom, abnormal assessment (laboratory test value, vital signs, electrocardiogram finding, etc.), or any combination of these.

AEs will be labeled according to severity (i.e., mild, moderate or severe), which is based on the AE's impact on the participant.

AEs will be categorized according to the likelihood that they are related to the study intervention or other study procedures (i.e., definitely unrelated, definitely related, probably related, or possibly related).

SAEs that are unanticipated, serious, and possibly related to the study intervention will be reported to the IRB, and NIMH in accordance with requirements. Unexpected fatal or life-threatening AEs related to the intervention will be reported to the NIMH Program Officer within 7 days. Other serious and unexpected AEs related to the intervention will be reported to the NIMH Program Official within 15 days. Anticipated SAEs will be handled in a less urgent manner but will be reported to the IRB in accordance with their requirements.

v. Statistical Software

All analyses will be performed using SAS and/or R.

vi. References

none
