# Supplementary material for: Effect of Enhanced Medical Rehabilitation on Functional Recovery in Older Adults Receiving Skilled Nursing Care After Acute Rehabilitation: A Randomized Clinical Trial
Source: JAMA Netw Open. 2019 Jul 31;2(7):e198199. doi: 10.1001/jamanetworkopen.2019.8199 (PMC6669784; doi:10.1001/jamanetworkopen.2019.8199)
Supplement: Supplement 2. — eTable. Barthel Changes by Individual Item in the Enhanced Medical Rehabilitation (EMR) and Standard of Care Groups [file jamanetwopen-2-e198199-s002.pdf]

Supplementary Online Content

Lenze EJ, Lenard E, Bland M, et al. Effect of enhanced medical rehabilitation on functional recovery in older adults receiving skilled nursing care after acute rehabilitation: a randomized clinical trial. *JAMA Netw Open*. 2019;2(7):e198199. doi:10.1001/jamanetworkopen.2019.8199

eTable. Barthel Changes by Individual Item in the Enhanced Medical Rehabilitation (EMR) and Standard of Care Groups

This supplementary material has been provided by the authors to give readers additional information about their work.

© 2019 Lenze EJ et al. *JAMA Network Open.*

eTable. Barthel Changes by Individual Item in the Enhanced Medical Rehabilitation (EMR) and Standard of Care Groups

Barthel item	Total			EMR			SOC		
	Baseline	Discharge	Time x Treatment	Baseline	Discharge	Main Effect of Time	Baseline	Discharge	Main Effect of Time
	(N = 228)	(N = 222)		(n = 114)	(n = 112)		(n = 114)	(n = 110)	
Are you able to feed yourself? (0-10)	6.82 (0.20)	8.09 (0.18)	F[1,223.89] = 0.01, p = 0.93	7.06 (0.26)	8.35 (0.23)	F[1,112.35] = 22.23, p < 0.001	6.58 (0.30)	7.83 (0.28)	F[1,111.52] = 17.86, p < 0.001
Are you able to bathe yourself? (0-5)	0.04 (0.03)	0.97 (0.13)	F[1,220.58] = 2.10, p = 0.15	0.04 (0.04)	1.16 (0.20)	F[1,111.02] = 31.91, p < 0.001	0.04 (0.04)	0.77 (0.17)	F[1,110.13] = 16.36, p < 0.001
Are you able to groom yourself? (0-5)	0.66 (0.11)	2.48 (0.17)	F[1,221.37] = 1.83, p = 0.18	0.48 (0.14)	2.54 (0.24)	F[1,111.70] = 72.64, p < 0.001	0.83 (0.18)	2.41 (0.24)	F[1,109.87] = 37.79, p < 0.001
Are you able to dress yourself? (0-10)	2.50 (0.18)	6.50 (0.20)	F[1,223.63] = 3.01, p = 0.08	2.37 (0.24)	6.78 (0.27)	F[1,112.30] = 179.52, p < 0.001	2.63 (0.25)	6.22 (0.28)	F[1,111.33] = 110.18, p < 0.001
Are you continent of your bowels? (0-10)	6.28 (0.32)	8.94 (0.20)	F[1,224.82] = 3.43, p = 0.07	6.01 (0.45)	9.28 (0.24)	F[1,112.71] = 56.46, p < 0.001	6.55 (0.45)	8.60 (0.32)	F[1,112.29] = 16.52, p < 0.001
Are you continent of your bladder? (0-10)	6.59 (0.30)	8.27 (0.25)	F[1,224.18] = 1.13, p = 0.29	6.32 (0.42)	8.31 (0.35)	F[1,112.97] = 21.57, p < 0.001	6.87 (0.42)	8.24 (0.35)	F[1,111.07] = 11.55, p < 0.001
Are you able to use the toilet by yourself? (0-10)	2.94 (0.17)	6.78 (0.20)	F[1,221.71] = 5.61, p = 0.02	2.63 (0.24)	7.01 (0.29)	F[1,112.08] = 159.04, p < 0.001	3.25 (0.23)	6.54 (0.27)	F[1,109.51] = 133.42, p < 0.001
Are you able to move from your bed to a chair and back? (0-15)	7.50 (0.20)	11.17 (0.20)	F[1,224.21] = 11.18, p = 0.001	7.19 (0.31)	11.65 (0.29)	F[1,112.68] = 154.44, p < 0.001	7.81 (0.25)	10.68 (0.28)	F[1,111.05] = 85.21, p < 0.001
Are you able to move about independently? (0-15)	0.18 (0.09)	8.17 (0.41)	F[1,220.19] = 0.57, p = 0.45	0.18 (0.12)	8.48 (0.59)	F[1,111.04] = 202.84, p < 0.001	0.18 (0.12)	7.86 (0.58)	F[1,109.19] = 175.72, p < 0.001

© 2019 Lenze EJ et al. *JAMA Network Open.*

Are you able to walk up and down stairs independently? (0-10)	0.02 (0.02)	3.88 (0.19)	F[1,220.02] = 1.02, p = 0.31	0.04 (0.04)	3.70 (0.30)	F[1,111.01] = 158.79, p < 0.001	0.00 (0.17)	4.05 (0.17)	F[1,113.99] = 280.63, p < 0.001
Statistics are Estimated Marginal Means and standard errors; the higher the score, the more independence participants demonstrated in completing the task.									

© 2019 Lenze EJ et al. *JAMA Network Open.*
