# Supplementary material for: Effect of Enhanced Medical Rehabilitation on Functional Recovery in Older Adults Receiving Skilled Nursing Care After Acute Rehabilitation: A Randomized Clinical Trial
Source: JAMA Netw Open. 2019 Jul 31;2(7):e198199. doi: 10.1001/jamanetworkopen.2019.8199 (PMC6669784; doi:10.1001/jamanetworkopen.2019.8199)
Supplement: Supplement 3. — Data Sharing Statement [file jamanetwopen-2-e198199-s003.pdf]

Data Sharing Statement

Lenze. Effect of Enhanced Medical Rehabilitation on Functional Recovery in Older Adults Receiving Skilled Nursing Care After Acute Rehabilitation. *JAMA Netw Open*. Published July 31, 2019. 10.1001/jamanetworkopen.2019.8199

Data
Data available: Yes
Data types: Deidentified participant data
How to access data: email: lenarde@wustl.edu
When available: With publication

Supporting Documents
Document types: None

Additional Information
Who can access the data: researchers whose proposed use of the data has been approved
Types of analyses: for a specified purpose
Mechanisms of data availability: without investigator support, after approval of a proposal, and with a signed data access agreement
Any additional restrictions: none
